# Excitation-transcription coupling in skeletal muscle: the molecular pathways of exercise

**DOI:** 10.1111/j.1469-185X.2010.00161.x

**Published:** 2011-08

**Authors:** Kristian Gundersen

**Affiliations:** Department of Molecular Biosciences, University of OsloP.O. Box 1041, Blindern, N-0316 Oslo, Norway

**Keywords:** skeletal muscle, exercise, plasticity, contraction speed, atrophy, hypertrophy, fiber type

## Abstract

Muscle fibres have different properties with respect to force, contraction speed, endurance, oxidative/glycolytic capacity etc. Although adult muscle fibres are normally post-mitotic with little turnover of cells, the physiological properties of the pre-existing fibres can be changed in the adult animal upon changes in usage such as after exercise. The signal to change is mainly conveyed by alterations in the patterns of nerve-evoked electrical activity, and is to a large extent due to switches in the expression of genes. Thus, an excitation-transcription coupling must exist. It is suggested that changes in nerve-evoked muscle activity lead to a variety of activity correlates such as increases in free intracellular Ca^2+^ levels caused by influx across the cell membrane and/or release from the sarcoplasmatic reticulum, concentrations of metabolites such as lipids and ADP, hypoxia and mechanical stress. Such correlates are detected by sensors such as protein kinase C (PKC), calmodulin, AMP-activated kinase (AMPK), peroxisome proliferator-activated receptor *δ* (PPAR*δ*), and oxygen dependent prolyl hydroxylases that trigger intracellular signaling cascades. These complex cascades involve several transcription factors such as nuclear factor of activated T-cells (NFAT), myocyte enhancer factor 2 (MEF2), myogenic differentiation factor (myoD), myogenin, PPAR*δ*, and sine oculis homeobox 1/eyes absent 1 (Six1/Eya1). These factors might act indirectly by inducing gene products that act back on the cascade, or as ultimate transcription factors binding to and transactivating/repressing genes for the fast and slow isoforms of various contractile proteins and of metabolic enzymes. The determination of size and force is even more complex as this involves not only intracellular signaling within the muscle fibres, but also muscle stem cells called satellite cells. Intercellular signaling substances such as myostatin and insulin-like growth factor 1 (IGF-1) seem to act in a paracrine fashion. Induction of hypertrophy is accompanied by the satellite cells fusing to myofibres and thereby increasing the capacity for protein synthesis. These extra nuclei seem to remain part of the fibre even during subsequent atrophy as a form of muscle memory facilitating retraining. In addition to changes in myonuclear number during hypertrophy, changes in muscle fibre size seem to be caused by alterations in transcription, translation (per nucleus) and protein degradation.

## Contents

- Introduction…………565
  1. Muscle fibre phenotypes…………565
  2. Changes in muscle fibre phenotypes…………566
  3. Determinants of muscle fibre phenotype…………567
  4. The importance of cell lineage…………567
- Cell external signals…………567
  1. What are the signals from the nerve?…………567
  2. The importance of nerve-evoked muscle activity…………568
    - The effects of normal and mismatching activity patterns on muscle contractile properties…………568
    - Dissecting the patterns…………569
  3. Mechanical stress…………570
- Intracellular signals…………571
  1. Calcium…………571
    - The source of elevated [Ca^2+^]_i_ during activity…………571
    - How does [Ca^2+^]_i_ fluctuate with different activity patterns?…………572
    - Calcium sensors: decoding the calcium fluctuations…………572
      - Calmodulin and its targets CaMKII and calcineurin…………572
      - Protein kinase C…………574
    - Cascades downstream of [Ca^2+^]_i_…………575
      - Ras andMAPK…………575
      - Protein kinase D…………575
      - Histone deacetylase…………576
      - PGC-1α and—β…………576
    - Transcription factors downstream of [Ca^2+^]_i_…………577
      - NFAT…………577
      - Does NFAT bind to fast or slow promoters?…………578
      - MEF2…………579
      - Myogenin and MyoD: the yin and yang of muscle plasticity?…………579
      - MyoD in slow-to-fast transformations…………580
      - Myogenin as a glycolytic-to-oxidative transforming agent…………581
  2. Metabolic activity correlates…………582
    - AMP-kinase…………582
    - PPARδ…………582
    - Oxygen…………583
  3. Six 1 and eya 1…………584
  4. MusTRD…………585
- Plasticity of muscle force…………585
  1. The cell biology of muscle fibre size…………585
  2. Paracrine and autocrine mechanisms…………587
    - Myostatin…………587
    - Insulin-like growth factor I (IGF-1)…………587
  3. Intracellular pathways of size regulation…………588
    - Regulation of protein production in muscle fibres…………588
      - Regulation of transcription…………588
      - Regulation of translation: the PI(3)K/Akt pathway…………588
    - Regulation of protein degradation…………588
    - Putative mechano-sensing mechanisms in muscle size regulation…………589
- The excitation-transcription coupling: towards a synthesis?…………589
  1. Plasticity of speed…………589
  2. Plasticity offorce…………590
- Conclusions…………590
- Acknowledgements…………590
- References…………590

## I. INTRODUCTION

The ability of adult muscle fibres to change in response to external stimuli has been called muscle plasticity. Force, contraction speed, endurance and oxidative/glycolytic capacity are all examples of muscle properties that are plastic. Skeletal muscle is a permanent, post-mitotic tissue, and unless the muscle is damaged there is little turnover of cells (Bintliff & Walker, 1960; Moss & Leblond, 1970, 1971; Stockdale & Holtzer, 1961). Thus, it has been demonstrated that dramatic changes in gene expression, protein composition and physiological properties can occur in pre-existing fibres without de- or regeneration (Delp & Pette, 1994; Gorza *et al.*, 1988; Mayne *et al.*, 1993; Windisch *et al.*, 1998). The plastic changes occur mainly by reprogramming the cell by turning on and off sets of relevant genes.

### (1) Muscle fibre phenotypes

**Fig. 1:**
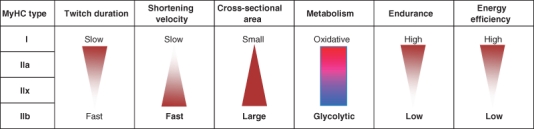
The properties of the four major fibre types found in mammalian muscle. There are exceptions to this general scheme. MyHC, myosin heavy chain.

It has long been known that skeletal muscles differ in phenotype (Ranvier, 1874) and there is a tendency for sets of properties to be connected, or clustered which has led to the notion of distinct muscle fibre types. The notion of type was supported by the observation that different myosin heavy chain (MyHC) ATPases have different pH optima allowing histochemical procedures to display a checkerboard-like staining pattern with distinct black and white fibres observed in cross sections (Brooke & Kaiser, 1970). A more modern approach has been to categorize muscle fibre type based on immunohistochemical labeling of distinct MyHC isoforms (Schiaffino *et al.*, 1989). The foundation for muscle fibre typing has been reviewed previously (Pette & Staron, 2000, 2001; Schiaffino & Reggiani, 1996), but a summary of fibre-type properties of limb muscles and the current fibre-type nomenclature is given in Fig. 1.

Originally, fibres were classified into three types based on histochemistry: I, IIa and IIb, with an additional IIc as a hybrid form. In rodents a fourth form was subsequently identified based on monoclonal antibodies against MyHC (Schiaffino *et al.*, 1989). This unknown “X” was dubbed IIx and is also called IId by some authors. In humans, fibres which had previously been called IIb express a MyHC gene that turned out to be homologous to the IIx gene in rodents (Ennion *et al.*, 1995; Smerdu *et al.*, 1994). Hence human fibre types are currently classified as I, IIa and IIx, where IIx is the former IIb described in older literature. The human genome also contains a gene homologous to the rodent IIb gene (Weiss, Schiaffino & Leinwand, 1999), but so far protein expression from this gene has not been demonstrated. In addition to MyHC, a large number of other contractile and structural proteins also come in distinct isoforms for which expression is more or less tightly connected to fibre type (Schiaffino & Reggiani, 1996). In addition to the three or four major MyHC genes expressed in adult limb muscles there are specialized forms expressed during development and in gill-arch-derived muscles (Hoh, 2002). In total 10 different MyHC genes have been connected to skeletal muscle (Desjardins *et al.*, 2002).

Analysis of physiological properties such as strength, twitch speed and endurance of single motor units suggests that units can be categorized into distinct largely non-overlapping groups correlating with their histochemical properties (Burke, 1967; Burke *et al.*, 1974; Burke, Levine & Zajac, 1971; Dum *et al.*, 1982); but the physiological properties of units of the same histochemical type can differ among muscles (Burke, 1967). In addition, although fibres belonging to one motor unit are all of the same MyHC type, measurements at the motor unit level might underestimate heterogeneity at the single fibre level. For example it has been demonstrated that both the shortening velocity and the MyHC composition might vary along the length of the fibre (Edman, Reggiani & Kronnie, 1985). While histochemical or immunohistochemical staining might give the impression that the vast majority of fibres are positive only for one MyHC, single-fibre gel electrophoresis has revealed that 11–67% of the fibres from various limb muscles express more than one MyHC isoform even under steady-state activity conditions (Stephenson, 2001). It can be concluded that the concept of universal fibre types throughout the body is an oversimplification.

The molecular foundation for the variability in contractile properties is partly known. For example, the MyHC isoform determines shortening velocity (the sliding velocity between actin and myosin), but also the ratio between the myosin light chain 3f and 2f isoform has a major influence. This ratio varies widely among individual fibres of the same MyHC type giving rise to rather large variability in shortening velocity within the type (Bottinelli & Reggiani, 2000; Schiaffino & Reggiani, 1996). Twitch speed is dependent on shortening velocity, but also on Ca^2+^ sequestering systems such as parvalbumin and the sarcoplasmic reticulum Ca^2+^ ATPases (SERCAs) (Gundersen *et al.*, 1988). Isoforms of SERCA are differentially expressed in different fibre types, thus SERCA2a is the predominant form in type I fibres while SERCA1a is expressed mainly in type II fibres (Periasamy & Kalyanasundaram, 2007). Although muscle endurance and fatigue seems to rely on many cellular factors (Allen, Lamb & Westerblad, 2008), endurance is correlated to high oxidative capacity with a high content of mitochondria and oxidative enzymes, and such properties are again linked to MyHC fibre type (Fig. 1).

### (2) Changes in muscle fibre phenotypes

The physiological properties (shortening velocity, twitch duration, endurance, etc.) that are linked in a fibre type are related to highly different molecular families (MyHC, SERCA, metabolic enzymes, etc.). As discussed below, coupling regulation of different physiological properties may be beneficial from an energy conservation point of view, and/or it might reflect common signaling systems diverging to regulate several sets of diverse genes. To some extent however, different properties can be uncoupled and regulated independently during plastic changes. For example, some degree of uncoupling has been observed between twitch speed and shortening velocity (Eken & Gundersen, 1988). More importantly, endurance-exercise in man and in other animals can lead to pronounced changes in metabolic properties without MyHC fibre-type conversion (Henriksson & Hickner, 1994; Saltin & Gollnick, 1983; Fitts & Widrick, 1996), although exercise can also change MyHC type in particular within type II (e.g. from type IIb/IIx to IIa) (Abdelmalki *et al.*, 1996; Andersen, Klitgaard & Saltin, 1994; Jansson *et al.*, 1990; Kraemer *et al.*, 1995; Staron *et al.*, 1994; Wang *et al.*, 1993), and under more severe experimental conditions fibre-type conversions are frequent.

When fibre-type conversions occur, it usually happens in a sequential order (Pette & Staron, 2000; Schiaffino & Reggiani, 1996): I ↔ IIa ↔ IIx ↔ IIb. During transitions hybrids between the “nearest neighbour” fibre type in this flow chart (e.g. I+IIa, IIa+IIx) are common, but aberrant hybrids such as I+IIb, I+IIa+IIb and I+IIx+IIb can also be seen under some experimental conditions (Caiozzo, Baker & Baldwin, 1998).

### (3) Determinants of muscle fibre phenotype

This review will discuss the factors that determine the molecular make-up of already formed adult muscles, and how that make-up can change. At any point in time, a fibre's make-up appears to depend on previous: (1) cell history/lineage; (2) nerve-evoked electrical activity; (3) mechanical conditions; (4) para-/autocrine conditions; and (5) circulating hormones.

Muscle properties are strongly influenced by hormones such as testosterone and thyroid hormones, as reviewed previously (Kadi, 2008; Pette & Staron, 2001; Staron *et al.*, 2000). This review will focus on the link between external factors related to activity and usage (points 2 and 3 above) and gene expression. The ability to change is, however, constrained by the muscle's cell lineage.

### (4) The importance of cell lineage

Developmental studies suggest that initial fibre-type differentiation might be determined by myoblast cell lineage independent of external influences such as innervation or usage. Thus, in experiments with quail chick chimera, limb myoblast diversity arises prior to the migration of myoblasts into the limb (Van Swearingen & Lance-Jones, 1995), and fibre-type distribution resembling the normal pattern is displayed even if the nerve is absent during development (Butler, Cosmos & Brierley, 1982; Condon *et al.*, 1990; Phillips & Bennett, 1984). When mammalian muscles are transplanted and made to regenerate in a different body location, some of the information determining fibre type is apparently derived from the muscle of origin rather than from the new position or innervation (Gutmann & Carlson, 1975; Hoh & Hughes, 1991; Pin *et al.*, 2002; Pin & Merrifield, 1997).

*In vitro* avian myoblasts constitute clones that give rise to specific fibre types (DiMario, Fernyak & Stockdale, 1993; DiMario & Stockdale, 1997; Miller & Stockdale, 1986), and when satellite cells derived from single mouse fibres form new myotubes, these express a MyHC type reminiscent of the fibre from which the satellite cells were derived (Rosenblatt, Parry & Partridge, 1996; but see also Bonavaud *et al.*, 2001). In adult rats, when different muscles are regenerating from myoblasts after myofibre destruction, the various regenerated muscles express different MyHC types reminiscent of the muscle of origin. This happens even if the muscles receive similar experimental patterns of activity. Thus, when regenerating soleus and extensor digitorum longus (EDL) were stimulated by the same slow pattern, the EDL failed to express the large amount of slow MyHC that was observed in the soleus under the same conditions (Kalhovde *et al.*, 2005).

It can be concluded that muscle fibre pedigree matters, and that there is a cell line component to the resulting adult phenotype of a fibre. The relationship is, however, not simple, since experiments with genetically marked myoblasts suggest that single myoblast clones can contribute to both fast and slow fibres, clones are not restricted to contribute to subsets of fibre types, and clones show no detectable preference for fusion to a particular fibre type (Hughes & Blau, 1992).

## II. CELL EXTERNAL SIGNALS

While it seems clear that cell lineage limits the adaptive range of muscle plasticity, it is equally clear that external signals can change muscle phenotype in the adult. In particular signals from the nerve appear to be important.

### (1) What are the signals from the nerve?

The field of muscle plasticity began with the seminal paper of Buller, Eccles & Eccles (1960) who showed that when a nerve from a fast muscle is transplanted to a slow muscle, and *vice versa,* both the reinnervated muscles change phenotype according to the new nerve supply. Subsequently, several other studies have confirmed this principle (reviewed by Close, 1972).

There were originally two theories for how the nerve could influence the muscle. The preferred hypothesis at the time suggested that different trophic substances were released from nerves supplying fast and slow muscles. But it was also recognized that differences in the pattern of action potentials in fast and slow motor units somehow could contain a coded message for muscle fibre change (Buller *et al.*, 1960).

While there is detailed information about the differences in the activity pattern of fast and slow motor units (Hennig & Lømo, 1985; Lømo, 2009), searches for relevant neurotrophic substances have failed. As discussed in detail in a previous review, there is little direct evidence for the existence of such factors regulating muscle contractile properties (Gundersen, 1998; but see Salviati, Biasia & Aloisi, 1986). In fact, stimulation of denervated muscles with patterns of activity resembling the activity in fast and slow motor units mimic virtually all the effects of cross innervation (Eken & Gundersen, 1988; Windisch *et al.*, 1998).

The old concept of neurotrophic (from the Greek *trophos* = nourishing) substances was based on the observation that denervation led to severe atrophy, but stimulating denervated muscles with implanted electrodes largely restores strength even after long-term denervation in both rats (Hennig & Lømo, 1987), and humans (Boncompagni *et al.*, 2007). Although the stimulated muscles never regain normal strength, activity is comparable to self-reinnervation in restoring maximal tetanic force production (Hennig & Lømo, 1987).

The converse experiment: to compare denervation to removal of action potential activity with an otherwise intact nerve supply, has led to more controversial results. Isolation of the spinal cord leads to less severe atrophy than denervation in the medial gastrocnemius and tibialis anterior muscles (but not in the soleus) (Hyatt *et al.*, 2003, 2006). These findings have been claimed as evidence for an important non-activity source of neural control (Roy *et al.*, 2008). It is however not unlikely that the difference is rather caused by small amounts of residual activity elicited in the isolated spinal cord. Stimulation of denervated muscles has shown that small amounts of electrical activity have a strong effect on muscle properties. For example a brief 0.6 s long high-frequency pulse train delivered every 100 min led to a sixfold increase in tetanic force of denervated muscles (Westgaard & Lømo, 1988). Moreover, in contrast to the experiments with spinal isolation, nerve impulse block with tetrodotoxin applied to the nerve, but with otherwise intact axons, induced muscle mass loss, fibre atrophy and reduction in force quantitatively indistinguishable from those induced by complete nerve transsection in rats (Buffelli, Pasino & Cangiano, 1997). It should be noted that extraordinary precautions were required to ensure complete absence of nerve-evoked activity even in this model.

In conclusion, there is currently no compelling evidence to suggest that there are any relevant sources of neural influence on the muscle other than activity, and in spite of intense searching for several decades, no neurotrophic substances have been identified that prevent atrophy or mimic other effects of normal innervation or cross-innervation outside the synaptic zone.

### (2) The importance of nerve-evoked muscle activity

#### (a) The effects of normal and mismatching activity patterns on muscle contractile properties

There are distinct differences in the pattern of activity evoked in fast and slow muscles (Hennig & Lømo, 1985). Generally type I motor units seem to receive high amounts of impulses delivered in long low-frequency trains, while type II units seem to receive short bursts of high-frequency activity. The total amount of impulses delivered to type II units is lower, but the amount seems to vary among the type II subtypes (Table 1).

**Table 1:** Firing characteristics of motor units in rats. Data are from Hennig & Lomo (1985) and represent the range of the means for 5–6 units followed over 24 h

Assumed fibre type	Instantaneous frequency (Hz)	Number of impulses in 24 hrs.	Number of impulses per train	% time active	Longest train duration
I	18–21	309 500–495 800	5–10	22–35	5–9 min
IIa or IIx	41–71	89 500–243 100	3–39	1.6–5.0	1.5–2.4 min
IIb	67–91	2 600–11 200	3–13	0.04–0.22	0.83–3.9 s

The importance of electrical activity has been confirmed by a large number of studies subsequent to the pioneering work of G. Vrbová and T. Lømo. Vrbová and collaborators demonstrated the importance of activity by stimulating muscles *via* the nerve (Vrbová, 1963), while Lømo's group studied denervated muscles and demonstrated the importance of muscle activity *per se* in the absence of any nerve influence (Lømo, Westgaard & Dahl, 1974).

In nerve-stimulation studies large amounts of low-frequency activity have been superimposed on normal background activity, leading to a fast-to-slow transformation. D. Pette and collaborators have, in a series of papers, described these changes extensively at the physiological and molecular/biochemical level (for reviews, see Pette & Vrbová, 1999, 1992). Since during nerve stimulation, the exogenous activity is superimposed on activity from the central nervous system, this limits pattern control. In particular, since the external activity is always added, the effect of a reduced amount of activity cannot be studied in innervated muscles. In slow muscles the large amounts of background activity will dominate, and preclude a study of slow-to-fast transformation. In addition, nerve stimulation does not preclude the possibility that the activity effects are secondary to changes in the motor neurone, for example *via* the release of putative neurotrophic substances (Gundersen, 1998).

An ideal model is to block nerve impulse activity with tetrodotoxin, and then stimulate the inactive nerve below the block (Ekmark *et al.*, 2007). The model is however technically demanding, and most of what we know about the importance of the pattern of activity is derived from stimulating denervated muscles directly. In such experiments pattern specificity has been demonstrated. If a pattern mimicking the native activity of a fibre type is used, more or less normal properties are maintained, while a mismatching pattern induces changes: a fast pattern leads to a slow-to-fast transformation in slow muscles; a slow pattern induces a fast-to-slow transformation in fast muscles. The physiological/molecular transformation encompasses metabolic properties/oxidative enzymes (Gundersen *et al.*, 1988), twitch duration/Ca^2+^-ATPase activity (Lømo *et al.*, 1974; Westgaard & Lømo, 1988), and shortening velocity/MyHC type (Ausoni *et al.*, 1990; Eken & Gundersen, 1988; Gorza *et al.*, 1988; Gundersen & Eken, 1992; Gundersen *et al.*, 1988; Schiaffino *et al.*, 1988, 1989; Windisch *et al.*, 1998).

#### (b) Dissecting the patterns

Simply mimicking the complex differences in the firing characteristics of fast and slow motor units (Table 1) does not provide information about which aspect of the differences is important, and more detailed experiments have been designed to unravel the “Morse code”.

**Fig. 2:**
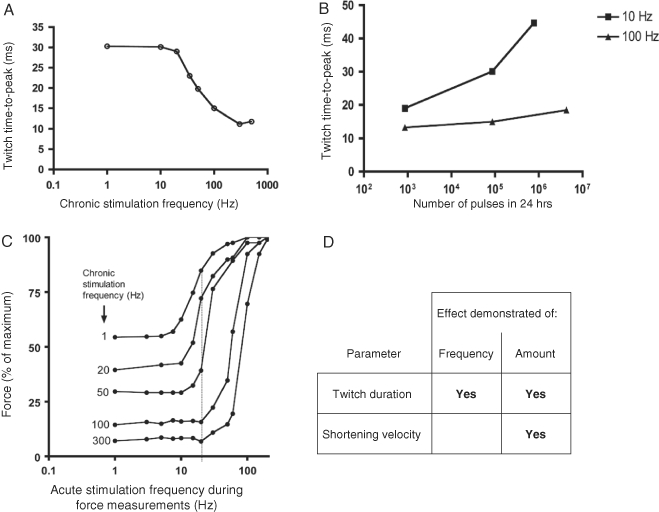
The effects of different chronic stimulation patterns on contractile properties of rat muscle. Twitch time-to peak seems to be regulated both by frequency (A), and amount (B). Teleologically, a frequency dependence of the twitch duration might ensure a match between the prevailing frequency received by the muscle and the frequency range that is efficient in regulating force by summation (C). In C note how the steep part of the force frequency curve moves towards the stimulation frequency. See for example how the force of an unfused tetanus with 20 Hz stimulation (broken vertical line) is on the steep portion of the curve for muscles receiving 20 Hz chronic stimulation. The data are replotted from Westgaard & Lømo (1988). (D) Summary of the dependence of different parameters on frequency and amount of chronic stimulation [for further details see text, Gundersen (1998) and Gundersen & Eken (1992)].

The most comprehensive study is by Westgaard & Lømo (1988). They studied the effects of different patterns of activity on isometric contraction. In particular, they showed a tight frequency dependence of twitch duration when the total number of impulses was kept constant. Thus, the twitch time-to-peak of the soleus fell from about 30 ms to 11 ms when the instantaneous frequency was increased from 1 Hz to 300 Hz (Fig. 2A). There is a good teleological explanation for the frequency dependence of the twitch: the twitch determines the fusion frequency of the tetanus, and in order to ensure efficient force regulation, the steep portion of the force-frequency curve should be matched to the firing frequency of the motorneurone. As can be seen in Fig. 2C, the steep portion of the curve moved towards the stimulation frequency. In addition to the frequency effect it was shown that when the frequency was kept constant at 10 or 100 Hz, respectively, higher amounts of stimuli led to slower twitches (Fig. 2B). This amount effect has led some authors to challenge the importance of frequency (Al-Amood & Lewis, 1987; Donselaar *et al.*, 1987; Kernell, Donselaar & Eerbeek, 1989). A distinct effect of frequency was demonstrated however; when 100 Hz bursts of stimulation were superimposed on a 10 Hz pattern, the twitch speed was reduced compared to the effect of the low-frequency pattern alone (Westgaard & Lømo, 1988). Thus, the high-frequency bursts made the twitch faster in spite of adding to the total amount of activity.

The twitch duration does not directly reflect sliding velocity between actin and myosin or MyHC type, as it is also heavily dependent on the kinetics of Ca^2+^ handling. There is much less information on the effects of electrical activity on isotonic shortening velocity or MyHC content. In a study where the train duration and the repetition rate of the trains (but not total number of impulses) was kept constant and the instantaneous frequency varied, low amounts of 20 Hz stimulation were as efficient in increasing shortening velocity of the soleus muscle as high-frequency patterns (Gundersen & Eken, 1992). Considering the effects of all the various activity patterns published there is no positive evidence that shortening velocity or MyHC composition is dependent on instantaneous frequency, and the amount of stimulation seems to be the important factor (Gundersen, 1998).

In conclusion, as summarized in Fig. 2D, the twitch duration seems to be controlled by both frequency and amount of activity, while there are no data demonstrating that frequency is important for shortening velocity or MyHC composition. Teleologically it would seem beneficial if muscles that are used frequently become more energy efficient. Both longer twitches and slower MyHC type contribute to higher energy efficiency for comparable amounts of external work (Gibbs & Gibson, 1972; Wendt & Gibbs, 1973).

Since the amount of activity seems to play a vital role, it seems important to determine what aspect of amount is of consequence. Amount has several attributes such as: (1) number of pulses per contraction; (2) duration of each contraction; (3) total number of pulses over long time periods (e.g. 24 h); (4) fraction of time a muscle remains contracted; and (5) duration of rest periods. Since these parameters do not change independently, it has been difficult to dissect the decisive factor, but for maintenance of normal fast contractile properties in the EDL, the number of impulses per train/contractile event (attribute 1) seems to be important for regulating shortening velocity and hence probably also MyHC type (Gundersen & Eken, 1992). This effect must somehow be decoded by a molecular impulse “counter” registering the number of impulses per activity episode, or if attribute 3 is important registering the number of impulses over longer periods of time. If attributes such as the fraction of time occupied by activity (4), the duration of rest periods (5) or the duration of each contractile event (2) are important, there must be a molecular “timer” rather than a “counter”. As discussed below, calcium-dependent kinases and phosphatases might serve as such “counters” or “timers” of nerve-evoked action potentials.

### (3) Mechanical stress

Action potential activity leads not only to depolarization and triggering of intracellular signals downstream of the depolarization, but also leads to shortening and/or mechanical tension. It is widely assumed that contraction against a resistance leads to larger muscles than contraction against lower resistance, but this does not necessarily have a direct bearing on the importance of mechanical factors as such. Thus, both the recruitment and the activity pattern of each motor unit vary with the force output. One model that has been used in attempts to manipulate mechanical and electrical factors more independently is hind limb suspension. This is a procedure where rats are chronically lifted by the tail so as to unload the hind limbs leading to atrophy. Initially, there is a halt in electrical activity as judged by an integrated electromyogram (EMG). The integrated EMG however appears gradually to recover to normal levels within a few days whereas muscle atrophy continues to progress (Alford *et al.*, 1987). This could mean that action potential activity and downstream events such as for example calcium release is of relatively little importance, and that a more important role should be postulated for force generation *per se*. This conclusion is however based on integrated EMGs only. Detailed information about firing properties of single motor units during hind limb suspension is not available, and, at least with intact feedback from proprioreceptors, it seems highly unlikely that the activity pattern in limbs that are not developing force should be identical to those of muscles exerting normal external force. Thus, hind limb suspension is not an optimal model for separating effects of mechanical stress and electrical activity.

The most compelling evidence for a mechano-dependent mechanism comes from experiments where limbs have been immobilized by a cast. This leads to atrophy, but studies over almost 100 years have shown that atrophy can be partly counteracted when muscles are immobilized in a lengthened position rather than a shortened position (Booth, 1977; Ferguson, Vaughan & Ward, 1957; Fournier *et al.*, 1983; Froboese, 1922; Goldspink, 1977; Herbert & Balnave, 1993; Kurakami, 1966; Meyer, 1922; Ralston, Feinstein & Inman, 1952; Savolainen *et al.*, 1988; Tabary *et al.*, 1972; Tardieu *et al.*, 1969; Thomsen & Luco, 1944; Yang *et al.*, 1997).

There are also studies suggesting that muscle length influences contraction speed such that chronic stretch makes a muscle slower; immobilization of fast muscles in a lengthened position thus increases the fraction of slow fibres (Goldspink, 1999; Goldspink *et al.*, 1992, 1991; Loughna *et al.*, 1990; Pattullo *et al.*, 1992). Similarly, overload elicited by ablation of synergists leads to pronounced changes in the slow direction (Gregory *et al.*, 1990; Gregory, Low & Stirewalt, 1986; Ianuzzo *et al.*, 1989; Ianuzzo, Gollnick & Armstrong, 1976; Kandarian, Schulte & Esser, 1992; Morgan & Loughna, 1989; Noble, Dabrowski & Ianuzzo, 1983; Periasamy *et al.*, 1989; Roy *et al.*, 1985; Tsika, Herrick & Baldwin, 1987). Again, the effect of length or load in these experiments could be secondary to the effect that these procedures have on the activity pattern. In fact, the properties of the motorneurones are dependent on muscle length, as it has been shown that the duration of the after-hyper-polarization, which is correlated to firing frequency, can be influenced by the length at which the muscles are immobilized (Gallego *et al.*, 1979). Moreover, integrated EMG measurements indicated that the amount of activity is lower when the muscle is immobilized in the shortened position (Fournier *et al.*, 1983; Hnik *et al.*, 1985). Thus, the apparent effects of length and load could be largely secondary to activity changes and changes in motor neurons rather than to mechanical factors.

A few length studies have been performed on denervated muscles showing that immobilization in the stretched position (Cotlar, Thrasher & Harris, 1963; Summers & Hines, 1951; Loughna & Morgan, 1999; Savolainen *et al.*, 1988) or tenotomy of synergistic muscles (Herbison, Jaweed & Ditunno, 1975; Schiaffino & Hanzlikova, 1970) counteracts atrophy in absence of the nerve. Moreover there is a minor counteracting effect of mechanical stretch on denervation atrophy, and similarly in tissue culture stretching myotubes increases protein synthesis (Vandenburgh & Kaufman, 1979) and decreases proteolysis (Vandenburgh & Kaufman, 1980).

Attempts have been made to train rat muscles by standardized electrical nerve stimulation, but letting the muscle contract under eccentric, isometric or shortening isotonic conditions. After eight weeks of training there were, however, no clear effects of the mechanical conditions on muscle mass (Adams *et al.*, 2004), but levels of some signaling substances such as myostatin and insulin-like-growth factor 1 (IGF-1) varied (Heinemeier *et al.*, 2007).

In summary, in most experimental conditions it is hard to separate electrical activity and mechanical stretch, but some experimental data point to the presence of an activity-independent mechanical mechanism influencing muscle size and perhaps contraction speed. The quantitative importance of mechanical mechanisms still seems somewhat elusive, and more studies where both activity and force are controlled separately should be performed.

## III. INTRACELLULAR SIGNALS

**Fig. 3:**
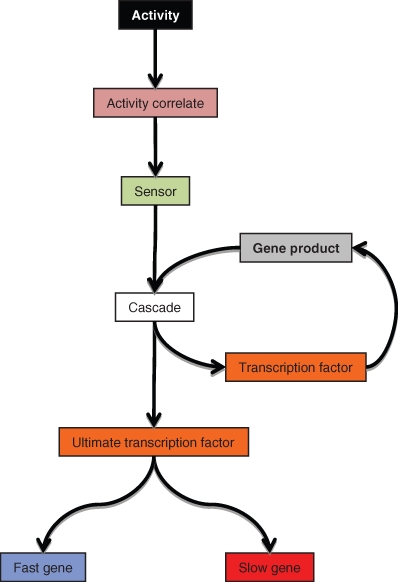
A simplified flow chart illustrating the excitation-transcription coupling, i.e. the flow of information from change in activity to change in fibre-type-specific gene expression.

There is a consensus that changes in muscle usage will transform muscle phenotype, but the precise biological signaling mechanisms responsible for such changes are less clear. A simplistic flow chart for how activity information could be processed by the muscle fibre is given in Fig. 3. As discussed above, muscle activity is important, and signaling pathways must somehow be triggered by an activity correlate. The relative importance of various activity correlates is not known, and in fact many factors may serve as “messengers”, such as: (1) free intracellular Ca^2+^ caused by cell membrane influx or release from the sarcoplasmatic reticulum; (2) metabolites (lipids, ADP, etc.); (3) hypoxia; and (4) tension (mechanosensation).

Few attempts have been made to dissect the various activity correlates, and it is hard to manipulate one parameter without affecting the others, but evidence related to each of these activity correlates that might serve as messengers will be discussed below.

The activity correlate must somehow be registered *via* a sensor that can transduce the correlate into a signal in an intracellular signaling cascade pathway. A cascade allows amplification and complex interactions with other pathways. A cascade might involve transcription factors acting on genes taking part in the signaling, but ultimately a pathway would end in affecting one or more ultimate transcription factors binding to the promoters of fast and slow genes. It should in fact be emphasized that transcription factors can act indirectly by regulating genes for various factors and thus taking part in the cascade (Fig. 3). Therefore there has been a quest for the transcription factors working at the end of the signaling pathway by binding selectively to promoters for fast and slow genes, such factors will be called ‘ultimate transcription factors' in this review.

### (1) Calcium

#### (a) The source of elevated [Ca^2+^]_i_ during activity

Free intracellular Ca^2+^ (Ca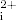) is the most abundant and potent of all second messengers, and action potential activity in muscle probably leads to the strongest Ca^2+^ release [mainly from the sarcoplasmic reticulum (SR)] that occurs in any cell type under physiological conditions. This has made fluctuations in cytosolic [Ca^2+^]_i_ the prime candidate for mediating the effects of electrical activity on muscle phenotype. Although the release from the SR is massive, there is also an influx across the cell surface, and the relative role of these two sources is not clear. For instance, it has been shown in organ culture that electrical activity stabilizes the acetylcholine receptors at the neuromuscular junction by way of an influx of ions through dihydropyridine-sensitive Ca^2+^channels in the sarcolemma, while release from the SR is ineffective (Caroni *et al.*, 1993; Rotzler, Schramek & Brenner, 1991). On the other hand, caffeine application leading to release of calcium from the SR elevated the level of several proteins regulated by activity *in vivo* such as glucose transporter type 4 (GLUT4) and oxidative enzymes (Ojuka, 2004; Ojuka *et al.*, 2002). More research would be required to establish the relative importance of different sources of Ca^2+^, and the possible importance of compartmentalization of [Ca^2+^]_i_ in regulating different plastic properties.

#### (b) How does [Ca^2+^]_i_ fluctuate with different activity patterns?

Early studies indicated that a slow stimulation pattern induced a sustained increased level of resting [Ca^2+^]_i_ in muscle fibres (Sréter *et al.*, 1987), More recently however, it was shown that short-term electrical stimulation (<24 h) of myotubes in culture did not induce a sustained increased [Ca^2+^]_i_, in spite of inducing expression of mRNA for slower MyHC types (Kubis *et al.*, 2003). Thus, slow activity patterns seem to be able to induce changes before sustained increased levels of Ca^2+^ are detected, and more acute fluctuations in [Ca^2+^]_i_ time-locked to the slow activity pattern are probably more important.

In resting mammalian muscle, various reports have indicated [Ca^2+^]_i_ ranging from 26 to 145 nmol l^−1^ (Konishi, 1998). In isolated mouse flexor digitorum brevis fibres, which were most likely of type II, fura-2 measurements indicate resting levels of 30–50 nmol l^−1^ (Westerblad & Allen, 1991). With slow stimulation at 10 Hz, mean [Ca^2+^]_i_ showed a modest increase to below 500 nmol l^−1^ in isolated fibres of mouse fast-twitch flexor digitorum brevis and slow-twitch soleus muscles (Aydin *et al.*, 2008; Westerblad & Allen, 1993). With a fast pattern at 100 Hz mean tetanic [Ca^2+^]_i_ increased to >1000 nmol l^−1^ (Allen *et al.*, 2008). Thus, it seems clear that both amplitude and temporal differences in the [Ca^2+^]_i_ signals downstream of different activity patterns might serve as a signal for changing gene transcription. There is currently however, no definitive evidence for such a role under physiological conditions in adult muscle *in vivo*. Future research should answer questions such as: what is the key source or relevant compartment for the [Ca^2+^]_i_ fluctuations? Moreover, the transients that are connected to specific fast and slow patterns that are known to induce changes in the fast and slow direction, respectively, should be mapped, and the differences between them should be connected to downstream signaling cascades that might regulate fibre-type-specific genes.

#### (c) Calcium sensors: decoding the calcium fluctuations

As concluded above, the rise in [Ca^2+^]_i_ connected to action potentials is a likely signal for muscle plasticity, but how are the signals decoded (for a previous review, see Buonanno & Fields, 1999)? The rather tight connection between that pattern of activity and variables such as the twitch time-to-peak suggest the existence of rather precise timing or counting mechanisms. Likely primary sensors for the signal are compounds such as protein kinase C and calmodulin.

##### (i) Calmodulin and its targets CaMKII and calcineurin

Calmodulin activates Ca^2+^/calmodulin-dependent protein kinase-II (CaMKII). CaMKII has been demonstrated to be activated during exercise (Rose & Hargreaves, 2003), but its function in synaptic plasticity in nerve cells is better characterized. In this intensely investigated field of neurobiology, CaMKII is thought to be involved in spike-timing-dependent plasticity. A central hypothesis has been that peak [Ca^2+^]_i_ determines the plasticity outcome, such that a sufficiently high [Ca^2+^]_i_ level leads to long-term potentiation (LTP) of the synapse, while moderately increased [Ca^2+^]_i_ leads to long-term depression (LTD) (Bi & Rubin, 2005). By analogy, in muscle a fast activity pattern leads to short-lived but high peak [Ca^2+^]_i_, while slow activity leads to sustained but more moderate levels, and these might turn on a fast and slow gene expression program, respectively. In addition to the importance of the peak levels of [Ca^2+^]_i_ that are discussed below, the rate of rise of [Ca^2+^]_i_ during the onset of an activity event might play a role (Buonanno & Fields, 1999).

In the brain, CaMKII is activated by the Ca^2+^/calmodulin complex, such that half-maximal activation occurs at a [Ca^2+^]_i_ of 500–1000 nmol l^−1^ (Rostas & Dunkley, 1992). In addition to the dependence on peak [Ca^2+^]_i_ there is a time-dependent component. Thus pulse exposure of CaMKII to Ca^2+^*in vitro* at room temperature showed that the enzyme reacted differently when Ca^2+^ pulses were delivered at 1 or 4 Hz or when the Ca^2+^ pulse duration was varied between 80 and 1000 ms (De Koninck & Schulman, 1998). Thus CaMKII might decode not only [Ca^2+^]_i_ levels, but also the temporal pattern of [Ca^2+^]_i_ fluctuations at a timescale relevant for decoding fast and slow patterns of motor activity.

**Fig. 4:**
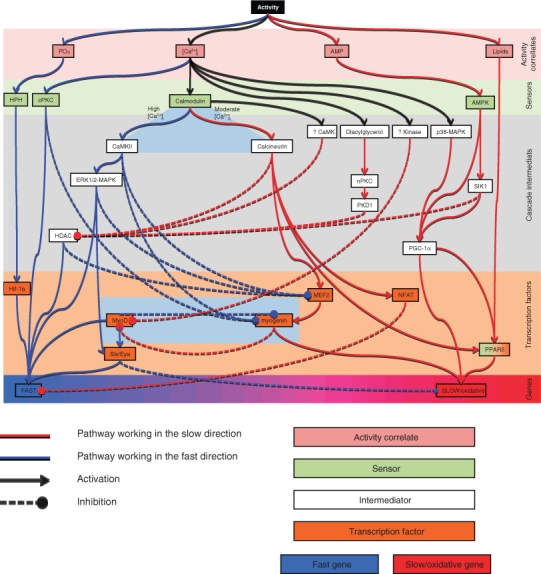
Diagram summarizing pathways currently believed to be involved in excitation-transcription coupling in skeletal muscle with respect to regulating contraction speed. The pathways have different degrees of scientific support, and their relative quantitative importance is still poorly understood. Abbreviations: adenosine monophosphate (AMP), AMP-activated kinase (AMPK), Ca^2+^/calmodulin-dependent protein kinase (CaMK), conventional protein kinase C (cPKC), extracellular signal-regulated kinase (ERK), free intracellular calcium concentration [Ca^2+^]i, HIF-1*α* prolyl hydroxsylases (HPH), histone deacetylase (HDAC), intracellular oxygen pressure (PO2i), mitogen-activated kinase MAPK, myocyte enhancer factor 2 (MEF2), myogenic differentiation factor (MyoD), novel protein kinase C (nPKC), nuclear factor of activated T-cells (NFAT), peroxisome proliferator-activated receptor *γ* (PPAR*γ*) coactivator-1*α* (PGC-1*α*), hypoxia inducible factor 1*α* (HIF-1*α*), peroxisome proliferator-activated receptor *δ* (PPAR*δ*), protein kinase D1 (PKD1), salt inducible kinase 1 (SIK1), sine oculis homeobox 1/eyes absent 1 (Six1/Eya1).

For LTD in the brain calcineurin also seems to be required (Mulkey *et al.*, 1994), and as discussed below calcineurin has been implicated in the fast-to-slow shift in fibre type. Like CaMKII, calcineurin is activated by calmodulin, but at much lower [Ca^2+^]_i_ levels because calmodulin has a much higher affinity for calcineurin (dissociation constant *K*_d_ = 0.1nmol l^−1^) than for CaMKII (*K*_d_ = 45nmol l^−1^) (Cohen & Klee, 1988). Hence, at moderate [Ca^2+^]_i_ the phosphatase calcineurin would be active (Crabtree, 1999), while at higher [Ca^2+^]_i_ (or at a higher frequency of the Ca^2+^spikes) the kinase CaMKII would also be active. The difference in the phosphorylation/dephosphorylation of substrates downstream of these two enzymes may trigger fast and slow gene transcription, respectively. In combination, calmodulin, CaMKII and calcineurin could decode both amplitude and temporal aspects of [Ca^2+^]_i_ (blue triangle in Fig. 4).

Calcineurin knock-out mice show a reduction in the number of slow fibres (Parsons *et al.*, 2003), and similar observations were made in mice where the knock-out was targeted to skeletal muscle only (Parsons *et al.*, 2004) Similar results were obtained in mice that overexpressed the calcineurin-inhibiting protein Regulator of Calcineurin 1 (RCAN1) in muscle fibres from early in development (Oh *et al.*, 2005). In tissue culture, the calcineurin-inhibitor cyclosporin A has been shown to block up-regulation of endogenous slow MyHC (Higginson *et al.*, 2002), and overexpression of active calcineurin transactivates promoter-reporter constructs for slow genes (Chin *et al.*, 1998). This finding has been criticized in another study where a larger variety of promoters were investigated, and it was found that calcineurin selectively activated several muscle-specific promoters, but with no selectivity for slow genes (Swoap *et al.*, 2000). For the endogenous MyHC genes in culture however, calcineurin increases the expression of slow isoforms, without any effect on fast MyHC isoforms (Delling *et al.*, 2000). Similar effects have been observed in transgenic mice overexpressing calcineurin driven by the muscle creatine kinase (MCK) promoter conferring early expression in myotubes (Naya *et al.*, 2000; Wu *et al.*, 2001). In regenerating muscle of wild-type mice the calcineurin inhibitors cyclosporin A, FK506 and Cain/cabin-1 prevent up-regulation of slow MyHC induced by slow patterned activity from motorneurones or electrodes (Serrano *et al.*, 2001).

Experiments in culture, transgenic mice or regenerating muscle such as those described above may reflect developmental effects, rather than adult plasticity. In adult mice, systemic administration of cyclosporin A increased the proportion of slow fibres in one study (Chin *et al.*, 1998), but failed to do so in another (Biring *et al.*, 1998). Moreover, cyclosporine has systemic effects on the animals' activity level, so the effects may be secondary to activity changes.

Overexpression of the calcineurin inhibitor cain/cabin-1 in adult rat soleus fibres after somatic gene transfer by electroporation led to the appearance of transcripts for type IIb and IIx MyHC, which is normally rare in this muscle. The transfected fibres expressing these MyHCs did not express type I or IIa MyHC, which are normally the most common forms in this muscle, implying that calcineurin activates these genes (Serrano *et al.*, 2001). These data support the idea that calcineurin is maintaining normal slow properties in adult slow muscles. Calcineurin may also be involved in fibre-type transformation in fast muscles since fast-to-slow transformation induced by overload was impaired when calcineurin blockers (FK506, cyclosporin A) were used (Dunn, Burns & Michel, 1999). Similar observations were made in calcineurin-deficient transgenic mice (Parsons *et al.*, 2004).

As discussed below, nuclear factor of activated T-cells (NFAT) is an important substrate for calcineurin, but it should be emphasized that NFAT is by no means the only protein dephosphorylated by this phosphatase. Knocking out two different variants of the calcineurin catalytic subunit A (A*α* and A*β*) both led to a dramatic reduction in slow fibre type and oxidative enzymes. However, only the A*α*-null mouse displayed reduced NFAT activity. This suggests the existence of a relevant calcineurin-activated pathway that is not NFAT dependent (Parsons *et al.*, 2003).

In conclusion, calcineurin may act in slow signaling both dependent on and independently of NFAT. Its effects might be both in activating slow genes, and in inhibiting fast genes (see Fig. 4).

##### (ii) Protein kinase C

The mammalian protein kinase C (PKC) family can be grouped into three classes: conventional (cPKC; *α*, *γ*, *β*I and *β*II), novel (nPKC; *δ*, *ε*, *η*/L, *θ*), and atypical (aPKC; ***ζ*, *ι*/*λ***). PKC ***μ*** and ***ν*** have been considered to be a fourth class but are now classified as protein kinase D (see Section III.1*dii*). PKCs are activated by translocating to the plasma membrane where they can be activated by Ca^2+^ and lipids. cPKCs are in themselves Ca^2+^ sensors, but are also activated by diacylglycerol and phosphatidylserine. Other PKCs are not directly Ca^2+^ dependent, but nPKC is stimulated by diacylglycerol and phosphatidylserine, and aPKC by phosphatidylserine only (Newton, 2001).

In cultured mast cells, cPKC*γ* has been shown to translocate in response to Ca^2+^ spikes, and the kinetics suggest that it may serve as a decoding machine for patterned [Ca^2+^]_i_ oscillation. In order to activate cPKC, [Ca^2+^]_i_ should reach a threshold level of 400 nmol l^−1^ (Almholt *et al.*, 1999; Mogami *et al.*, 2003); and localization seems to be important since Ca^2+^ influx over the surface membrane seems to be much more efficient than release from internal stores (Mogami *et al.*, 2003; Pinton *et al.*, 2002). Muscle has however a massive Ca^2+^ release from its specialized endoplasmatic reticulum (SR), and the importance of this release has not been investigated. With respect to temporal aspects, in the timescale of seconds, high-frequency, but not low-frequency calcium spikes elicit high cPKC*γ* activity (Oancea & Meyer, 1998), suggesting that cPKC might be activated by fast, but not slow, patterns of activity (but see also Pedersen *et al.*, 2009).

In rat skeletal muscle PKC has been demonstrated to be translocated and activated by electrical stimulation *in vivo* (Cleland *et al.*, 1989; Huang, Tong & Schmidt, 1992), but cPKC did not seem to be affected by exercise in humans (Rose *et al.*, 2004). In developing avian muscle and also tissue culture, PKC seemed to be negatively regulated by nerve-evoked activity, thus PKC activity was decreased by innervation and increased by blocking neuromuscular transmission in slow muscles. In the avian models PKC activity was also found to be higher in fast than in slow muscles, and elevation of cPKC*α* and nPKC*θ* reduced expression of a slow MyHC isoform in slow muscles (DiMario, 2001; DiMario & Funk, 1999; Jordan *et al.*, 2004). In conclusion it could be speculated that various cPKCs might serve as a sensor for fast activity patterns mediating a suppression of slow MyHC in muscles receiving such activity (Fig. 4). However, although it has been demonstrated that a slow MyHC isoform can be regulated by PKCs, it remains to be demonstrated if they do so in adult muscle *in vivo*.

Among the novel PKCs, nPKC*θ* is the most abundant isoform in skeletal muscle (Chang *et al.*, 1993; Osada *et al.*, 1992), and the protein is 2.5-fold more abundant in fast than in slow muscles (Donnelly *et al.*, 1994). Although nPKCs lack the Ca^2+^-binding domain, it has been shown in neuro-endocrine cells that Ca^2+^ influx produced diacylglycerol that in turn translocates and activates nPKC*θ* over a timescale of seconds (Mogami *et al.*, 2003). nPKCs could hence act as pattern-specific signal decoders of nerve-evoked activity in muscle. Protein kinase D1 (PKD1) is an interesting downstream target for nPKC*θ* and other nPKCs such as nPKC***ε*** and nPKC***η*** (Rykx *et al.*, 2003; Yuan *et al.*, 2002). As illustrated in Fig. 4 and discussed in Section III.1*dii*, PKD1 would act in the slow direction, and thus nPKCs might have the opposite role as cPKCs.

Little is known about aPKCs in muscle, but activity may regulate aPKC *via* AMP-activated kinase (AMPK) (Chen *et al.*, 2002); aPKC isoforms are activated within minutes following the onset of a single bout of endurance exercise both in mice (Chen *et al.*, 2002) and humans (Nielsen *et al.*, 2003; Perrini *et al.*, 2004; Rose *et al.*, 2004). aPKC has not directly been implied in regulation of fast and slow genes, but interestingly, aPKC can activate mitogen-activated kinases (MAPKs) (Hirai & Chida, 2003), which might take part in such regulation. More research seems, however, required to clarify if aPKC has a role in muscle plasticity.

#### (d) Cascades downstream of [Ca^2+^]_i_

##### (i) Ras and MAPK

Ras is a subfamily of small GTP-binding proteins encoded by three different genes in mammals. Ras is localized to the inner face of the plasma membrane and functions as a molecular switch that transmits receptor signals, but it can also be regulated by pathways dependent on Ca^2+^ influx, including at least one calmodulin-dependent pathway. Downstream of Ras is a cascade involving mitogen-activated kinases (MAPKs) originally called extracellular signal-regulated kinases (ERKs). The MAPK family has many members such as eight ERKs (ERK1-8), p38 and Jun N-terminal kinase (JNK). The MAPKs are turned on by phosphorylation. Active MAPKs in turn have a multitude of downstream target genes (Bogoyevitch & Court, 2004; Bradley & Finkbeiner, 2002; Chang & Karin, 2001; Finkbeiner & Greenberg, 1996).

MAPKs are activated in a variety of endurance-type exercises and other activity models both in animals and in man (Kramer & Goodyear, 2007); in particular a slow, but not fast pattern increased ERK activity (Murgia *et al.*, 2000). Blocking of ERK1/2 with U0126 decreased type I and increased type II MyHC mRNA expression in primary culture (Higginson *et al.*, 2002). In regenerating slow muscles a constitutively active form of Ras and a MAPK both mimicked the effects of slow nerve activity, while a dominant negative Ras prevented nerve-evoked activity from inducing slow MyHC in regenerating muscle (Murgia *et al.*, 2000). Thus Ras and MAPK might be involved in maintaining slow properties in this model; but since active Ras was unable to induce slow myosin in fast regenerating muscle (Murgia *et al.*, 2000), Ras apparently does not take part in fast-to-slow transformation.

Findings in C2C12 tissue culture and in adult animals have, however, not supported the findings in regenerating muscle, as MAPKs rather seem to act in the fast direction. Thus, it was found that ERK1/2 activity was twofold higher in fast than in slow adult muscle (Shi *et al.*, 2008, 2007). In addition, while the levels of ERK1 and 2 were similar in soleus, ERK2 was the predominant form in the fast EDL. No differences were found with respect to p38 and JNK (Shi *et al.*, 2007), although these MAPKs are also activated by exercise (Kramer & Goodyear, 2007). In C2C12 cells in tissue culture when constitutively active ERK2 was overexpressed fast promoter-reporter genes such as *MyHCIIb* and *SERCA1* were activated. The ERK1/2 inhibitor PD98059 had the opposite effect, as it was shown that the MyHCI promoter was stimulated while the MyHCIIb and SERCA1 promoters were inhibited. Similarly, endogenous expression of fast MyHC and SERCA1 was decreased while the levels of MyHCI and myoglobin increased (Shi *et al.*, 2008).

In adults MAPK was inhibited by electroporation of an expression vector for MAPK phosphatase 1 (MKP1) which will dephosphorylate and inactivate ERK1/2. This procedure activated MyHCI promoter-reporter constructs, and reduced fast SERCA1 and MyHCIIb reporter levels; endogenous MyHC type I and IIa genes also were activated in fast IIx or IIb fibres. In slow soleus fibres, MyHC1 promoter-reporter genes were induced even more strongly than in fast muscles. When a constitutively active form of ERK2 was overexpressed in fast fibres it had no effect on fast or slow promoter-reporter constructs, but in the slow soleus MyHCIIb reporter level was increased, although MyHCI reporter was not decreased (Shi *et al.*, 2008). Thus, ERK2 seems to activate fast genes in slow muscles, while its role in inhibiting slow genes remains more uncertain.

In conclusion, manipulating MAPKs has given somewhat conflicting results in different models. During regeneration MAPK seems to promote slow properties, while in adult muscle fibres there is evidence that MAPKs can be involved in slow-to-fast transformation of pre-existing fibres. How this role could be related to the findings that slow activity increases MAPK activity (Kramer & Goodyear, 2007; Murgia *et al.*, 2000) remains unclear.

##### (ii) Protein kinase D

Protein kinase D1 (PKD1; previously also called PKC***μ***) is one of a three-member kinase family (Rykx *et al.*, 2003). PKD is not directly regulated by calcium, but can be activated through phosphorylation by nPKC isoforms such as PKC***ε***, PKC***η*** and PKC*θ* (Rykx *et al.*, 2003; Yuan *et al.*, 2002). PKD1 is enriched in slow fibres. Forced expression of a constitutively active form of PKD1 from a MCK promoter, which confers expression from early muscle differentiation, developed transgenic mice with perturbed fibre-type distribution, smaller fibres and a somewhat reduced fibre number. The fraction of type I fibres was increased, and the level of IIa and IIx mRNA and protein was increased, while IIb and IIx MyHC and mRNA did not change significantly. Myoglobin levels also increased (Kim *et al.*, 2008). On the other hand a muscle-specific knock-out of PKD1 had no effect on fibre types, but fatigability was increased (Kim *et al.*, 2008). In these experiments positive effects might be attributed to effects during development, and the lack of effects to compensatory mechanisms. Nonetheless, the observed perturbation of fibre type composition opens up the possibility that PKD1 is involved in regulating fibre type in adult animals.

PKD1 influences many fundamental cell biological processes including membrane trafficking, cell survival, differentiation and migration. The Raf–MEK–ERK pathway (MEK is MAP/ERK kinase) and nuclear factor of *κ* light polypeptide gene enhancer in B-cells (NF*κ*B) seem to be among the downstream targets it activates (Rykx *et al.*, 2003; Van Lint *et al.*, 2002). In cardiomyocytes PKD1 has been shown directly to phosphorylate histone deacetylase (HDAC) 5 and stimulate its nuclear export (Kim *et al.*, 2008). A hypothetical pathway where calcium influx leads to elevation of diacylglycerol activating nPKCs, activating PKD1, inhibiting HDACs, and thus acting in the slow direction is illustrated in Fig. 4.

##### (iii) Histone deacetylase

HDACs are a family of proteins with the ability to deacetylate a variety of proteins, not only histones. Thus, HDACs might influence gene expression not only by affecting chromatin structure but also by interacting with other transcription factors. The HDAC subclass IIa has the highest expression in brain and muscle, and has been implicated in muscle plasticity. The IIa class consists of HDAC4, −5, −7 and 9. Class IIa HDACs have low deacetylase activity and might not be authentic deacetylases, they can however function as co-repressors of transcription in interaction with transcription factors. They might also interact with class I HDACs to regulate histone acetylation (Mejat *et al.*, 2005). The class IIa HDACs are regulated not only transcriptionally but by several post-translational modifications including ubiquitination which leads to degradation, and phosphorylation which leads to nuclear export. Knock-out experiments suggest that the different class IIa HDACs can substitute for each other. HDAC biology was reviewed recently (Haberland, Montgomery & Olson, 2008; Walkinshaw *et al.*, 2008).

In spite of lower RNA levels, HDAC IIa protein levels are higher in fast muscles compared to slow, apparently due to ubiquitin-proteasome-mediated degradation acting selectively in slow muscles (Potthoff *et al.*, 2007). Knock-out mice lacking individual class IIa HDACs displayed no changes in muscle fibre type. However when double knock-outs of two HDAC IIa genes were created, the animals showed increased numbers of type I and IIa fibres, and elevated levels of mRNA for these slow MyHC types (Potthoff *et al.*, 2007). These data suggest that HDACs might be involved in fibre-type differentiation during development, but not necessarily in muscle plasticity in adults.

The importance of HDACs was also investigated in adult mice by overexpressing HDAC5 from an inducible promoter. When such animals were subjected to treadmill running, the elevated levels of HDAC5 prevented an exercise-induced increase in type I and IIa fibres in the plantaris muscle (Potthoff *et al.*, 2007). In isolated adult muscle fibres, it was shown that slow stimulation led to an export of HDAC4 (but not HDAC5) from the nucleus, and that this process was dependent on calmodulin kinases (Liu, Randall & Schneider, 2005).

These experiments suggest that HDACs somehow maintain fast properties, and that reduced HDAC signaling facilitates fast-to-slow transformation.

Signaling pathways coupling specific patterns of activity to HDAC activity are not well understood, but phosphorylation could provide a rapid activity-dependent response. Four putative inhibitory pathways acting on HDAC are illustrated in Fig. 4. (1) Experiments in C2C12 cells suggest that HDAC5 could be negatively regulated by AMPK by activating salt inducible kinase (SIK1) (Takemori *et al.*, 2008). (2) Forced expression of constitutive active forms of calcineurin using the MCK promoter in transgenic mice leads to development of an increased number of slow fibres which is paralleled by a decrease in HDAC5 and -7 (Naya *et al.*, 2000; Potthoff *et al.*, 2007), and this mechanism might be relevant also in adult muscle. (3) Similar results to those found with calcineurin have been obtained with CaMKIV (Wu *et al.*, 2002), but this kinase is not normally expressed in muscle (Bassel-Duby & Olson, 2006), so perhaps another CaMK is operating in this tissue? (4) Diacylglycerol might have an effect *via* PKD1.

##### (iv) PGC-1α and—β

Peroxisome proliferator-activated receptor *γ* (PPAR*γ*) coactivator-1*α* (PGC-1*α*) and its homolog PGC-1*β* are co-activators of transcription factors, and may be among the best-studied examples of an increasingly recognized group of proteins that regulate transcription without themselves having independent DNA-binding capability. The PGC-1 molecules have been implicated mainly in regulating pathways related to mitochondrial oxidative metabolism, and to glucose, lipid and energy homeostasis. PGC-1*α* is a co-activator not only for PPAR*γ* from which its name is derived, but also the other PPARs, including PPAR*δ* (Lin, Handschin & Spiegelman, 2005) which has been implicated in muscle plasticity (discussed in Section III.2b). For PGC-1*β*, co-activation of PPAR*α*, and—*γ* has been established but the ability to co-activate PPAR*δ* has not been determined. In addition to the PPARs, PGC-1*α* and—*β* also have other downstream targets; and for PGC-1*α* it has been established that forkhead box O1 (FoxO1), myocyte enhancer factor 2 (MEF2), cAMP response element-binding (CREB) and sex determining region Y-box 9 (Sox9) are among them (Lin *et al.*, 2005). As discussed below FoxO1 seems to be central for muscle size regulation, while MEF2 has been implicated in muscle plasticity, and another member of the *Sox* gene family (Sox6) has been implicated in the development of muscle fibre type (Hagiwara, Ma & Ly, 2005; Hagiwara, Yeh & Liu, 2007; Hofsten *et al.*, 2008).

PGC-1*α* is preferentially expressed in slow muscles both at the mRNA and protein level (Lin *et al.*, 2002*b*). In both humans and in rodents physical exercise increases PGC-1*α* levels (Atherton *et al.*, 2005; Baar *et al.*, 2002; Goto *et al.*, 2000; Norrbom *et al.*, 2004; Pilegaard, Saltin & Neufer, 2003; Terada *et al.*, 2002, 2005; Wright *et al.*, 2007). In tissue culture application of a Ca^2+^-ionophore increased PGC-1*α* levels. Both in culture and *in vivo* PGC-1*α* concentration is elevated by slow-patterned electrical stimulation (Irrcher *et al.*, 2003). The latter probably indicates that activity *per se* influences PGC-1*α* levels; in addition catecholamine (Miura *et al.*, 2007) and thyroid hormones (Irrcher *et al.*, 2003) have been implicated in up-regulating PGC-1*α* levels in muscle. Activity seems to regulate not only the level of PGC-1*α*, but also the translocation of the protein to the nucleus (Wright *et al.*, 2007).

A possible mediator for activity effects on PGC-1*α* is the MAPK p38, and interestingly, p38 activation seems to be related to mechanical stretch (Akimoto *et al.*, 2005; Boppart *et al.*, 2001; Goodyear *et al.*, 1996; Long, Widegren & Zierath, 2004; Martineau & Gardiner, 2001; Wretman *et al.*, 2001). In tissue culture, PGC-1*α* could also be regulated by SIK1 (Takemori *et al.*, 2008) and AMPK (Irrcher, Ljubicic & Hood, 2009; Irrcher *et al.*, 2008; Takemori *et al.*, 2008) and *in vivo* (Suwa, Nakano & Kumagai, 2003).

When PGC-1*α* was overexpressed in muscle using the MCK promoter in transgenic mice, slower muscles were developed, with higher levels of mitochondrial enzymes, and appearance of 10–20% type I fibres in muscles that normally contain almost exclusively type IIb fibres (Lin *et al.*, 2002*b*). Knock-out of PGC-1*α* had no clear effect on the development of MyHC fibre type, although oxidative capacity was reduced (Arany *et al.*, 2005). The results are however difficult to interpret since the mice were hyperactive. When a muscle-specific knock-out was made these animals developed a slow-to-fast shift in MyHC fibre type and a reduced oxidative capacity, thus these animals showed approximately the reverse change compared to animals overexpressing PGC-1*α*. The animals were however spontaneously hypoactive (Handschin *et al.*, 2007), and this might in itself lead to a shift in fibre type.

In conclusion there are indications that PGC-1*α* is involved in a slow phenotype. As hypothesized in Fig. 4, PGC-1*α* may be regulated by p38 and AMPK. PGC-1*α* might activate slow genes by acting as a PPAR*δ* co-factor, but could also act on other downstream targets independently of PPAR*δ*.

PGC-1*β* was cloned much later than PGC-1*α* (Lin *et al.*, 2002*a*) and there is less accumulated information. Its role also seems to be more complex, not fitting into a simple fast-to-slow or slow-to-fast transformation model. PGC-1*β* is not clearly differentially expressed in fast and slow muscles, but at least in some muscles, PGC-1*β* seemed to be associated with type IIx fibres (Arany *et al.*, 2007). Exercise has been reported either to have no effect on the level of PGC-1*β* (Meirhaeghe *et al.*, 2003) or to induce a decrease (Mathai *et al.*, 2008; Mortensen *et al.*, 2007). When PGC-1*α* or PGC-1*β* were overexpressed in muscle tissue culture they had similar effects; oxidative enzyme activity was increased and mRNA for MyHCI was increased while IIb and IIx were reduced; PGC-1*β* also led to up-regulation of IIa (Mortensen *et al.*, 2006).

PGC-1*β* was expressed in transgenic mice with the same MCK promoter as for PGC-1*α*, but with different results. In muscles that were developed in PGC-1*β* mice, there was no alteration in MyHCI, but IIa and IIb expression was suppressed. The IIx isoform was highly elevated in the fast/mixed muscles that were tested, and *in situ* hybridization suggested that IIx MyHC was highly expressed in nearly all fibres. PGC-1*β* also increased mitochondrial biogenesis and oxidative enzyme activity, and increased running distances to exhaustion for the mice (Arany *et al.*, 2007).

In conclusion, although overexpression of PGC-1*β* changed fibre type to the intermediate IIx form, its relationship to muscle activity remains unclear. Moreover, both for PGC-1*α* and PGC-1*β* it remains to be investigated what the role of the cofactor is in regulating adult properties. The effects observed so far have all been in transgenic animals where PGC-1 is present during part of muscle development.

#### (e) Transcription factors downstream of [Ca^2+^]_i_

##### (i) NFAT

Nuclear factor of activated T-cells (NFAT) is a five-gene family of transcription factors with multiple splicing variants expressed from each gene. NFATc1-4 are similar in structure and have been investigated in muscle (Lopez-Rodriguez *et al.*, 1999; Rao, Luo & Hogan, 1997).

In B-cells it was shown that low sustained levels of elevated [Ca^2+^]_i_ activates NFAT (Dolmetsch *et al.*, 1997), and a similar [Ca^2+^]_i_-calmodulin-calcineurin-NFAT pathway was subsequently suggested for the excitation-transcription coupling in muscle (Chin *et al.*, 1998). It is believed that calcineurin acts by dephosphorylating NFAT, which again triggers a translocation of NFAT into the cell nuclei where it can act as a transcription factor (Crabtree & Olson, 2002; Rao *et al.*, 1997). Re-phosphorylation of NFAT leads to export from the nucleus, and in muscle this seems to be mediated by glycogen synthase kinase 3*β* (GSK3*β*) or casein kinase 1 or 2 (CK1/2) (Shen *et al.*, 2007).

In order to establish NFAT as a substrate for calcineurin in the context of muscle plasticity, several groups have used the peptide VIVIT, which inhibits the calcineurin-NFAT interaction without affecting general calcineurin phosphatase activity (Aramburu *et al.*, 1999). In primary cultures, co-transfection with VIVIT blocked the positive effect of calcineurin on slow MyHC expression by 70%, but had no effect on fast MyHC. In adult slow muscle VIVIT was shown to block the expression of MyHCI, and was also shown to inhibit nerve-activity-induced expression of slow myosin in the regenerating soleus muscle (McCullagh *et al.*, 2004). These experiments suggest that calcineurin-NFAT interaction could be important in maintaining slow properties in the slow soleus muscle. The critical experiment to demonstrate that calcineurin-NFAT interaction is crucial for activity-induced fast-to-slow fibre-type transformation, however, would be to investigate if VIVIT can block the effects of slow stimulation in an adult fast muscle, but such experiments have yet to be reported.

NFATc1-4 are all expressed in skeletal muscle, but only NFATc1 (also called NFATc or NFAT2a, but not to be confused with NFAT1c) seems to be preferably translocated to the nuclei in slow fibres. Although NFATc4 and to some extent NFATc2 and NFATc3 also show nuclear localization in muscle, it is not fibre-type specific (Calabria *et al.*, 2009; Tothova *et al.*, 2006). Translocation seems to be regulated by activity; when slow muscles are inactivated for 2 h NFATc1 is exported from the nucleus (Kubis *et al.*, 2002; Liu *et al.*, 2001; Tothova *et al.*, 2006), while slow but not fast electrical stimulation translocates it to the nucleus (Calabria *et al.*, 2009; Shen *et al.*, 2006). NFATc2 and -3 are also to some extent translocated by activity but with no clear pattern specificity, NFATc4 is localized to the nuclei irrespective of activity (Calabria *et al.*, 2009).

In order to investigate NFAT transactivating activity directly, experiments have been performed *in vivo* with artificial promoters where a high number of NFAT binding sites were coupled to luciferase as a reporter. It was shown that the transactivating activity of NFAT was higher in slow than in fast muscle, and it was reduced in slow muscle subjected to denervation or a fast stimulation pattern (McCullagh *et al.*, 2004). This suggests that NFAT can take part in maintaining slow properties in slow muscles. It was not investigated if NFAT transactivating activity increases in fast muscles subjected to slow stimulation, hence NFATs role in fast-to-slow transformation was not directly addressed in these experiments.

A constitutively active form of NFATc1 has been overexpressed in various muscle models *in vivo* (McCullagh *et al.*, 2004). Slow MyHC was increased in denervated, regenerating soleus and EDL muscles, which normally do not express this isoform. When co-electroporated with promoter-reporter constructs in the intact adult fast EDL muscle, the MyHCI promoter was induced sixfold while the IIb promoter was completely inhibited (but see also Swoap *et al.*, 2000). For the endogenous genes in intact adult muscle, NFAT downregulated the IIb gene, but surprisingly no type I was induced (McCullagh *et al.*, 2004). This discrepancy illustrates that neither regenerating muscle nor episomal promoter-reporter genes necessarily represent gene regulation of endogenous genes in intact adult muscle.

Even if overexpression of NFATc1 failed to activate MyHCI expression in EDL after seven days of treatment (McCullagh *et al.*, 2004), a role in fast-to-slow transformation could not be excluded. Thus, during the first two weeks of slow stimulation IIb expression is inhibited, but with only a minor induction of MyHCI. Remarkably, massive MyHCI expression commenced only after prolonged stimulation (Ausoni *et al.*, 1990; Windisch *et al.*, 1998) suggesting different regulatory mechanisms in the long term. However, stimulation rapidly inhibited IIx expression and increased IIa expression in these experiments. Unfortunately the effects of overexpressing NFATc1 on these genes have not been reported.

Loss-of-function experiments with inhibitory RNA gainst NFATc1-4 have been performed *in vivo* (Calabria *et al.*, 2009). With promoter-reporter constructs it was shown that activation of the MyHCI promoter was reduced by reducing any of the NFATs. Reducing NFATc1 had no effect on any of the type II MyHCs, while reducing NFATc4 reduced all of the type II MyHCs. NFATc2 and -3 reduced transactivation of IIa and IIx MyHC, but had no effect on IIb. The positive effect of NFATc1-4 on MyHCI expression was confirmed for the endogenous gene, but only in regenerating muscle.

Based on all these experiments it is tempting to suggest that NFATc1 is the factor activating a slow expression program, while NFATc4 is activating a fast program. NFATc4, however is not expressed in a fibre-type-specific fashion, nor is it regulated by activity. While NFATc1 seems to downregulate the endogenous MyHCIIb in intact adult muscle, it remains to be seen if it can turn on endogenous MyHCI under such conditions, which would be the hallmark of a fast-to-slow transition.

##### (ii) Does NFAT bind to fast or slow promoters?

Effects of various transcription factors on fast and slow promoters might reflect direct binding to gene regulatory sequences of such genes (regulation in cis as ultimate transcription factors), or they might regulate other genes that in turn regulate the genes determining fast and slow phenotype (Fig. 3). The original idea of the calcineurin-NFAT pathway was that calcineurin was activated by a slow stimulation pattern, the calcineurin then dephosphorylated NFAT that in turn transactivated slow genes by NFAT binding directly to slow promoters (Chin *et al.*, 1998). Binding to relevant promoters, however, was not directly demonstrated in their study.

The promoters for the fast and the slow isoforms of troponin I are the best characterized of the fast and slow specific promoters and they have been used in the quest for the ultimate fast and slow transcription factors. Troponin I (Tn1) is the regulatory component of the troponin complex, and probably takes part in determination of twitch speed (Squire & Morris, 1998; Zot & Potter, 1987). In adults the fast and the slow gene are expressed in fast and slow muscle fibres, respectively (Hallauer & Hastings, 2002), and the expression can be modulated by electrical activity (Buonanno *et al.*, 1998; Calvo *et al.*, 1996; Rana, Gundersen & Buonanno, 2008; Rana *et al.*, 2005). In transgenic mice, slow-specific expression is conferred by a 128 base pair (bp) rat sequence from the TnI slow gene dubbed the slow upstream regulatory element (SURE), and fast-specific expression by a 144 bp sequence from the fast TnI in quail called the fast intronic regulatory element (FIRE) (Banerjee-Basu & Buonanno, 1993; Hallauer & Hastings, 2002; Nakayama *et al.*, 1996).

Putative NFAT binding sites have been identified in both FIRE and SURE. Constitutively active calcineurin was shown to activate SURE (but not FIRE) promoter-reporter constructs *in vitro*, and the response in SURE was attenuated by mutating the NFAT site (Wu *et al.*, 2000). *In vivo*, the situation has been less clear. In one study on transgenic animals differential expression in fast and slow muscles from a SURE-reporter construct was abolished by mutating the NFAT site (Wu *et al.*, 2000), while in a different study a SURE construct from which the NFAT site was removed continued to exhibit slow-specific expression (Calvo *et al.*, 1999). Thus, the role of NFAT as an ultimate transcription factor directly activating slow genes still remains somewhat uncertain.

By contrast, in FIRE, a NFAT binding site has been documented by site-directed mutations electromobility shift assays and supershift assays. By utilizing novel time lapse *in vivo* imaging techniques (Rana *et al.*, 2005) it was shown that the NFAT binding site was necessary for the suppression of the fast troponin I promoter by a slow stimulation pattern, while the destruction of the site had no effect on the positive regulation of this gene by fast activity (Rana *et al.*, 2008).

In conclusion, current evidence suggests that NFAT signaling directly or indirectly connects slow activity to maintaining slow properties in slow muscles, and that NFAT is an ultimate transcription factor in inhibiting expression of the fast isoform of troponin I, and perhaps MyHC IIb during fast-to-slow transformation. The identification of NFAT as an inhibitory transcription factor for fast troponin I, might be one of the few documented examples of an ultimate transcription factor involved in differential expression of fibre-type-specific genes (see also Section III.3).

##### (iii) MEF2

The myocyte enhancer factor 2 (MEF2) transcription family of MADS box transcription factors are encoded by four different genes in vertebrates denoted as A, B, C, and D, each of which diplay various splicing variants. MEF2 is important during early muscle development (reviewed in Potthoff & Olson, 2007). The number of type I fibres was reduced in MEF2c and MEF2d, but not in MEF2a knock-outs. When a hyperactive MEF2c-VP16 fusion protein was overexpressed in muscle driven by the myogenin promoter, the number of type I fibres developed was increased, and the mice displayed improved running endurance (Potthoff *et al.*, 2007). These experiments suggest that some of the MEF2 isoforms are implicated in establishing slow fibre identity during development.

In adult animals MEF2 protein is expressed at similar levels in fast and slow muscles (Potthoff *et al.*, 2007), but MEF2 activity can be modified by mechanisms not related to its concentration. Thus, several kinases can inactivate MEF2 by phosphorylation including calmodulin-dependent kinases and MAPK, and it can be activated by dephosphorylation by calcineurin (Mao *et al.*, 1999; Wu *et al.*, 2000). MEF2 also seems to be regulated through HDAC class IIa proteins. When HDAC is present in the nucleus, it will bind to MEF2 and repress its transactivating ability (reviewed in Bassel-Duby & Olson, 2006; Walkinshaw *et al.*, 2008).

In order to investigate MEF2 activity, a transgenic mouse was developed where the reporter lacZ is driven by triplicated MEF2 binding sites from the desmin promoter (Naya *et al.*, 1999). In most mice no lacZ expression was detected in the adult, but lacZ-staining was visible in some soleus muscles, suggesting that MEF2 activity was higher in this slow muscle than in fast muscles. When the transgenic animals were subjected to treadmill running, the lacZ reporter became strongly increased in the soleus and in some, but not all fast muscles in the running lower leg. These findings suggest that exercise activity increased MEF2 transactivating activity. The effect was suggested to be calcineurin dependent, since injection of the calcineurin inhibitor cyclosporine A prevented a slow pattern of electrical stimulation from inducing lacZ. Moreover, when transgenic mice expressing the calcineurin inhibitor myocyte-enriched calcineurin-interacting protein 1 (MCIP1) driven by the MCK promoter were crossed with the MEF2-lacZ reporter mice, the mice developed muscles where treadmill running did not induce lacZ expression (Wu *et al.*, 2001).

A cautionary remark should be made with respect to using lacZ as a reporter gene in muscle, as it has been shown that the level of lacZ protein is strongly influenced by muscle activity in adult muscle. Thus, in transgenic mice where lacZ is driven by a promoter for the acetylcholine receptor *α* subunit, denervation leads to a marked decrease in lacZ staining in spite of the lacZ mRNA being strongly up- regulated. It was suggested that inactivity strongly promotes lacZ protein degradation (Gundersen, Sanes & Merlie, 1993). In avian myotube cultures similar experiments to those described for lacZ, using luciferase as a reporter, revealed no difference between MEF2 activity in fast and slow muscle with or without innervation from a spinal cord explant (Jordan *et al.*, 2004). The pattern of activity imposed by the explants was, however, not characterized.

The SURE and FAST elements from the slow and fast troponin I promoters respectively can also be used to illuminate the role of the MEF2 binding site, which are necessary for transcription of both enhancers in skeletal muscle (Calvo *et al.*, 1996; Nakayama *et al.*, 1996). In co-transfection experiments in tissue culture, various forms of MEF2 generally up-regulated expression from SURE more than from FIRE, and this effect is strongly potentiated by simultaneously expressing a constitutively active form of calcineurin. Overexpression of calcineurin alone also had a selective effect on SURE, and this effect was abolished by mutating the SURE MEF2 site into the homologous site in FIRE. This site provides weaker binding of MEF2 in electromobility shift assays (EMSA). These findings led to the suggestion that activation of calcineurin in slow muscles, or muscles subjected to slow stimulation, dephosphorylates and activates MEF2, which binds to and transactivates the slow but not the fast troponin I promoter (Wu *et al.*, 2000). However, this hypothesis has not been supported by *in vivo* experiments (Calvo *et al.*, 1999, 2001). In transgenic mice harbouring SURE elements, slow fibre-type-specific expression (but not muscle specificity) was lost when elements upstream of the MEF2 site were removed. When homologous elements from FIRE were ligated upstream of the part of SURE containing the MEF2 site, high expression was conferred in fast muscles, demonstrating that fibre-type-specific expression was not conferred by the MEF2 site, but rather by other promoter elements in SURE. In other words, when the SURE MEF2 site was added to the FIRE sequence, FIRE dominated (Calvo *et al.*, 2001; Rana *et al.*, 2008). Thus, the MEF2 site seems not to confer fibre-type specific expression of the slow troponin isoform.

Even if these experiments suggest that MEF2 is not a ultimate factor activating slow genes, MEF2 might be involved in slow-specific expression by activating other transcription factors, thus it is interesting that MEF2 has been shown to transactivate the myogenin promoter in tissue culture (Edmondson *et al.*, 1992; Friday *et al.*, 2003). As discussed below, myogenin may in turn induce oxidative properties, but apparently not slow MyHC fibre type (Ekmark *et al.*, 2003).

##### (iv) Myogenin and MyoD: the yin and yang of muscle plasticity?

Myogenic differentiation factor (MyoD), myogenin, myogenic regulatory factor 4 (MRF4), and myogenic factor 5 (myf-5 ) are a group of proteins that are specific to skeletal muscle and belong to the basic-helix-loop-helix (bHLH) family of transcription factors. These four proteins are called the myogenic regulatory factors (MRFs), as they play a key role during early differentiation of muscle cells. Like other bHLH proteins they are thought to bind to DNA mainly as heterodimers with E-proteins. The dimers bind to E-boxes in the DNA with the canonical sequence CANNTG. Other partners for bHLH transcription factors are inhibition-of-DNA-binding-proteins (Id-proteins) that act negatively since the heterodimers do not bind to DNA. Thus, the activity of MRFs can be regulated by modifying the level of the MRF or one of its partners. In addition, the MRFs have phosphorylation sites that influence protein stability (Song *et al.*, 1998), or prevent DNA binding (Bengal *et al.*, 1994; Li *et al.*, 1992*b*). There is evidence that these sites can be phosphorylated by PKC and CaMKII (Blagden, Fromm & Burden, 2004; Li *et al.*, 1992*b*) but apparently not protein kinase A (PKA) (Li *et al.*, 1992*a*; Winter, Braun & Arnold, 1993). It is unclear if different MRFs are selectively phosphorylated by different kinases.

MyoD acts early in myogenesis to determine myogenic fate, while myogenin acts later in the differentiation of myoblasts into myotubes (Berkes & Tapscott, 2005; Buckingham *et al.*, 2003). MyoD is involved in chromatin remodeling when fibroblasts differentiate into myoblasts, and in this model MyoD seems to have little ability to transactivate genes independently. Myogenin on the other hand seems to act more as a conventional transcription factor (Bergstrom & Tapscott, 2001; Cao *et al.*, 2006; Davie *et al.*, 2007; Ohkawa, Marfella & Imbalzano, 2006). It should be kept in mind, however, that gene regulation even with the same transcription factors and genes, is different in tissue culture, during development, regeneration, and in the adult animal (e.g. see Davie *et al.*, 2007; Zammit *et al.*, 2008).

All the MRFs are expressed in adult muscle, although myf-5 only at very low levels. MRF4 is the factor with the highest concentration in adults. MRF4 seems to be predominantly expressed in slow/oxidative fibres in some, but not all muscles (Walters, Stickland & Loughna, 2000*a*). On the other hand, promoter elements conferring expression in fast muscle fibres have also been identified in the MRF4 promoter (Pin & Konieczny, 2002). Further investigation is required to establish if MRF4 might play a role in maintaining or regulating adult muscle fibre type.

In adults, MyoD and myogenin show reciprocal expression patterns in vertebrates as diverse as mammals and fishes. The MyoD level is high in fast muscles and myogenin level is high in slow muscles; in rodents MyoD cis regulatory regions seem to restrict expression of MyoD to IIx and IIb fibres (Delalande & Rescan, 1999; Ekmark *et al.*, 2007; Hughes *et al.*, 1993; Rescan, Gauvry & Paboeuf, 1995; Voytik *et al.*, 1993). Contributing strongly to the difference in MyoD activity between fast and slow muscles are the differences in T115 phosphorylation. Thus the amount of phosphorylated, inactive MyoD was three times higher in soleus than in EDL, and since the total MyoD level was about half, the fraction of the MyoD that is inactive must be six times higher in soleus than in EDL (Ekmark *et al.*, 2007).

*In vitro* experiments have suggested that the different MRFs regulate one other in a positive manner (Olson, 1990; Weintraub, 1993), but this might be different *in vivo*. Thus in transgenic mice starting to overexpress myogenin in differentiated muscle cells, MyoD expression is decreased (Gundersen *et al.*, 1995). In mice with reduced MyoD expression, myf-5 expression is increased in a dose-dependent manner (Rudnicki *et al.*, 1992). If MyoD and myogenin cross regulate each other negatively *in vivo*, such a coupling would tend to stabilize fibres as ‘MyoD’ or ‘myogenin’ fibres, respectively, and hence as distinct fibre types (blue rectangle in Fig. 4). Altered activity or forced expression of one of the factors would undermine the stability, and transform the phenotype.

In most exercise studies MyoD and myogenin mRNA or protein is found to be elevated both after resistance (Bickel *et al.*, 2005; Drummond *et al.*, 2008; Haddad & Adams, 2002; Hentzen *et al.*, 2006; Hulmi *et al.*, 2007; Ishido, Kami & Masuhara, 2004; Kosek *et al.*, 2006; Kvorning *et al.*, 2007; Liu *et al.*, 2008; McKay *et al.*, 2008; Peters *et al.*, 2003; Psilander, Damsgaard & Pilegaard, 2003; Raue *et al.*, 2006; Willoughby & Nelson, 2002; Yang *et al.*, 2005), and endurance (Kadi *et al.*, 2004; Siu *et al.*, 2004; Vissing *et al.*, 2008) training. A few studies that have investigated both factors find an elevation only in MyoD (Harber *et al.*, 2008; Yang *et al.*, 2005), or only in myogenin (Costa *et al.*, 2007; Siu *et al.*, 2004). Some studies report little change (Bamman *et al.*, 2004; Coffey *et al.*, 2006; Hulmi *et al.*, 2008a; Jensky *et al.*, 2007) or even a reduced level (Costa *et al.*, 2007; Hulmi *et al.*, 2008*b*).

Studies based on muscle homogenates are however hard to interpret. Resistance training in particular leads to focal muscle damage and repair, and myogenic factors are strongly elevated in regenerating muscle tissue (Fuchtbauer & Westphal, 1992; Mendler *et al.*, 1998). The MyoD and myogenin relevant to muscle plasticity would have to be present in the myonuclei of pre-existing fibres. Importantly, an immunohistochemical analysis indicated that one bout of endurance training led to accumulation of myogenin in some myonuclei. This type of exercise had no effect on MyoD levels (Kadi *et al.*, 2004). Overload exercise, on the other hand, elevated the level of both MyoD and myogenin in some myonuclei (Ishido *et al.*, 2004).

##### (v) MyoD in slow-to-fast transformations

Thyroid hormone treatment results in activation of both MyoD and fast MyHC gene expression in the slow soleus *in vivo* (Hughes *et al.*, 1993). The effect of fast stimulation on the MyoD protein content was investigated in the soleus muscle by blocking the endogenous slow impulse activity with tetrodotoxin, and then stimulating the nerve below the block with a fast pattern. After 14 days the average MyoD protein level was increased, but the change was not significant. When the fast rat EDL muscle with its high MyoD content was subjected to a slow pattern of electrical stimulation *via* the nerve, the level of MyoD was initially unaltered. Thus, although it was reduced after 14 days (Ekmark *et al.*, 2007), regulation of the total MyoD protein level does not seem to be important for the initial phenotypic changes obtained by electrical stimulation. With an antibody specific for inactive MyoD phosphorylated at T115, it was found that levels of phosphorylated and inactive MyoD protein were significantly increased already after 10 h of slow stimulation, and after 14 days of stimulation levels more than doubled. At this time the level of total MyoD was reduced to half, so stimulation increased the fraction of inactive MyoD by four times. The analysis did not allow a quantification of the amount of MyoD that is active. However, it was suggested that in a normal EDL, the fraction of inactive MyoD could not be higher than 1/4 of the total. These data suggest that dephosphorylated MyoD could take part in maintaining fast properties in fast muscle, and that inactivation of MyoD could take part in fast-to-slow transformations. It is less clear if activation of MyoD could take part in slow-to-fast transformation. Thus, when slow rat soleus was stimulated with a fast pattern for two weeks, the level of phosphorylated MyoD was virtually unchanged (Ekmark *et al.*, 2007).

MyoD-null mutant mice failed to display any major shift in MyHC expression, although subtle shifts in the fibre type of fast muscles towards a slower character were seen; in slow muscle a tendency towards a faster phenotype was observed (Hughes *et al.*, 1997; Seward *et al.*, 2001). The lack of change might however be attributed to compensatory mechanisms during development; in order to test the possibility that MyoD might act in adult muscle fibres of normal mice, MyoD was overexpressed in slow muscle fibres. Wild-type MyoD had rather mild effects on fibre type. This was attributed to the activity-dependent phosphorylation discussed above, as it was subsequently shown that after denervation, in the absence of slow activity, overexpression of wild-type MyoD increased the number of fibres displaying fast MyHC significantly in both mice and rats. In further experiments a T115A mutation was introduced, and when this constitutively active form of MyoD was overexpressed after electroporation, the number of MyHC type II positive soleus fibres increased from 50% to 85% in mice and from 13% to 62% in rats, in spite of the muscles receiving the slow endogenous activity pattern (Ekmark *et al.*, 2007).

Thus, active MyoD is a very potent activator of fast transcription in adult muscle, and it could act in three different ways: (1) indirectly by activating genes for other signaling molecules; (2) directly by chromatin remodeling; or (3) as an ultimate transcription factor acting on type II MyHC promoters and other fibre-type-specific promoters.

There are few data to illuminate whether or not MyoD is acting to remodel chromatin in adult post-mitotic muscle fibres, but in the context of MyHC the structural organization of MyHC genes seems suitable for such regulation: in both mice and man the slow MyHCI gene is located on chromosome 14, while the fast IIa, IIx, and IIb forms are clustered on a 300–600 kb segment in the order IIa-IIx-IIb on human chromosome 17 and mouse chromosome 11. Other multi-gene families of contractile proteins are however dispersed throughout the genome, and may require different regulatory mechanisms (Vikstrom *et al.*, 1997).

There is some evidence that MyoD could act as an ultimate transcription factor for MyHC genes. In mice, the IIa and IIb genes each have two E-boxes, while the IIx gene has seven (Allen *et al.*, 2001); but after overexpression of MyoD in C2C12 cells only the IIb, and not the IIa and IIx, promoter is activated (Allen *et al.*, 2001). This demonstrates that MyoD can work selectively on different MyHC promoters in myotubes in culture, and might provide a mechanism for MyoD maintaining IIb expression in normal EDL muscles. In the soleus, fibres overexpressing MyoD and MyHC IIa and IIx were induced, but no IIb was detected. Interestingly this mirrors the effect of fast-patterned electrical stimulation, which also turns on IIa and IIx myosin, while no MyHC IIb is detected in soleus even after more than two months of fast electrical stimulation (Ausoni *et al.*, 1990). Even cross-innervation for more than a year failed to produce shortening velocities consistent with a high type IIb fibre content (Close, 1969; Eken & Gundersen, 1988). Thus, *in vivo* there seem to be similar limitations for cross-innervation, electrical stimulation, and MyoD overexpression.

A differential effect of MyoD and myogenin has been discussed also for genes other than MyHC relevant to fibre type, and it has been suggested that the E-boxes flanking sequences are involved in determining target sequence specificity for different MRFs. In chicken the fast myosin light chain 1 (MyLC1) has an upstream regulatory region where the distal part contains two E-boxes which are acting as an enhancer responsive to MyoD, while the proximal part contains a single E-box responsive to myogenin (Asakura *et al.*, 1993).

In the fast troponin I gene from quail, the affinity of MyoD for various E-boxes was found to correlate with the ability to trans-activate, and substitution of E-boxes from other muscle-specific genes conferred less binding of MyoD, and less expression when introduced into the fast troponin I gene (Yutzey & Konieczny, 1992). On the other hand, for a chimeric construct combining the E-box parts of SURE with parts of FIRE not containing an E-box, the construct conferred fast expression in adult animals (Calvo *et al.*, 1999, 2001; Rana *et al.*, 2008). Thus, in these experiments the differences between the E-boxes or their flanking sequences were not important for the fibre-type-specific expression, suggesting that MyoD does not act as the ultimate fast transcription factor for TnI. There is more hope for MyHC, but further *in vivo* experiments are required to establish if MyoD is an ultimate transactivator for type II MyHC, or if it acts indirectly *via* other signaling systems or by chromatin remodeling.

##### (vi) Myogenin as a glycolytic-to-oxidative transforming agent

When fast rat EDL muscles are subjected to a slow pattern of electrical stimulation *via* the nerve, the level of myogenin protein and mRNA in the muscle is elevated after a few hours; conversely myogenin concentration is reduced in slow muscles upon fast stimulation (Ekmark *et al.*, 2007), or fast cross innervation (Hughes *et al.*, 1993). When fast muscles in hypothyroid rats are subjected to slow stimulation, myonuclei in fast-to-slow transforming fibres show accumulation of myogenin mRNA (Putman, Dusterhoft & Pette, 2000). Thus, changes in the myogenin protein level respond in a timely and pattern-specific fashion and could be responsible for changes in the slow/oxidative direction.

Myogenin knock-out mice die at birth (Hasty *et al.*, 1993; Nabeshima *et al.*, 1993), precluding their use in illuminating the role of myogenin in adult muscles. Mice overexpressing myogenin in muscle also had a high neonatal mortality, but for those that survived there were no changes in MyHC fibre type. The activity levels of oxidative mitochondrial enzymes in fast muscles were however increased two- to threefold, whereas levels of glycolytic enzymes were reduced to 30–60% of normal values. Histochemical analysis shows widespread increases in mitochondrial components and glycogen accumulation. The changes in enzyme content were accompanied by a reduction in fibre size, such that many fibres acquired a size typical of oxidative fibres (Hughes *et al.*, 1999). In a subsequent study myogenin expression vectors were injected intracellularly into single adult muscle fibres, or introduced by electroporation into a limited number of fibres. These experiments essentially gave the same results as those observed in the transgenic animals (Ekmark *et al.*, 2003), demonstrating that myogenin can alter the phenotype of pre-existing muscle fibres in adult mice.

Thus, myogenin seems to mimic the typical effects of endurance training since changes in metabolic properties without changes in MyHC type are observed after moderate endurance training in mammals, including humans (Fitts & Widrick, 1996; Henriksson & Hickner, 1994; Saltin & Gollnick, 1983), although changes in MyHC type at least within type II (e.g. from type IIb/IIx to IIa), can also occur (Abdelmalki *et al.*, 1996; Kraemer *et al.*, 1995; Wang *et al.*, 1993).

Like MyoD, myogenin has an inhibitory phosphorylation site (T87) that was shown to be phosphorylated by a high-frequency stimulation pattern (Blagden *et al.*, 2004), but more experiments would be required to establish pattern specificity. An interesting possibility would be if myogenin and MyoD were both phosphorylated by activity, but with a different pattern specificity.

Alterations in oxidative capacity are a question of regulating mitochondrial quantity and quality, and transcriptional regulation of mainly nuclear, but also mitochondrial genes is involved. In general, a large number of nuclear genes are regulated by a small number of transcription factors such as nuclear respiratory factor 1 (NRF-1) and NRF-2. These factors regulate nuclear genes for mitochondrial proteins, and expression of transcription factors regulating genes in the mitochondrial genome. Mitochondrial composition (Forner *et al.*, 2006; Mootha *et al.*, 2003) and relevant regulatory mechanisms (Scarpulla, 2008) are partially tissue specific. Thus, the muscle-specific protein myogenin could possibly work as an ultimate transcription factor binding directly to promoters of mitochondrial enzymes. Several of them contain the consensus sequence for the binding site of bHLH transcription factors (E-box), and in genes encoding muscle/heart-specific forms of the cytochrome c oxidase (COX) subunits VIII and VIa, it has been shown that an intact E-box is required for efficient tissue-specific transcription (Lenka *et al.*, 1996; Wan & Moreadith, 1995). For the COX VIII subunit, negative effects of Id-1 were also demonstrated, strengthening the idea that bHLH proteins are important in regulating this gene.

Interestingly the protein zinc finger protein 106 (ZFP106) is directly regulated by both myogenin and NRF-1 binding to its promoter. The exact role of ZFP106 is not known, but it has been implicated in insulin receptor signaling (Grasberger *et al.*, 2005).

Myogenin may also act indirectly by regulating other transcription factors such as the nuclear respiratory factors (NRFs). PGC-1*α* can act as a cofactor for NRF-1 and is thought to be crucial for the regulation of oxidative capacity (Lowell & Spiegelman, 2000; Scarpulla, 2008). PGC-1*α* in turn seems to be regulated by MRFs binding directly to the core PGC-1*α* promoter. The binding of myogenin to the relevant E-box is slightly stronger than that of MyoD, but in the *in vitro* assays tested so far myogenin did not have a stronger trans-activating ability than MyoD (Chang *et al.*, 2006).

### (2) Metabolic activity correlates

The availability of a variety of metabolites changes during activity. Two examples are ADP and lipids, and studies of signaling molecules that are sensors for ADP and lipids, respectively, suggest that these activity correlates can serve as messengers for muscle plasticity.

#### (a) AMP-kinase

Active muscles have elevated AMP/ATP ratios that may serve as an activity correlate sensed by AMP-activated kinase (AMPK). It is known that AMPK is activated by exercise; when the AMPK agonist aminoimidazole carboxamide ribonucleotide (AICAR) was administered to rats, metabolic genes were induced and running endurance was increased by 44% (Narkar *et al.*, 2008).

Interestingly, the AMPK*α* subunit co-immuniprecipitates with peroxisome proliferator-activated receptor *δ* (PPAR*δ*) (Narkar *et al.*, 2008) suggesting that this kinase might also interact directly with this lipid sensor.

#### (b) PPARδ

PPARs are ligand-activated transcription factors of the nuclear receptor superfamily. Lipids such as free fatty acids (FFAs) serve as ligands for these receptors, and hence PPARs are lipid sensors. In the current context PPARs therefore combine the role of sensors and transcription factors.

FFAs are elevated in active muscles and might activate the PPARs during activity (Nawrocki & Gorski, 2004). Another interesting possibility would be if metabolites from food intake in itself may influence relevant signaling systems. Changes in fibre type and metabolic properties have been reported after feeding experiments (Nakazato & Song, 2008), but it can not be excluded that the effects are secondary to activity changes.

Three mammalian subtypes of PPARs, -*α*, -*γ*, and -*δ* (the latter also called -*β*), have been identified (Dreyer *et al.*, 1992; Kliewer *et al.*, 1994). The PPARs form dimers with retinoid X receptors (RXRs). PPAR*α* and PPAR*γ* are predominantly expressed in liver and adipose tissue, respectively, while PPAR*δ* is the predominant isoform in heart and skeletal muscle (Braissant *et al.*, 1996; Escher *et al.*, 2001; Kliewer *et al.*, 1994; Muoio *et al.*, 2002).

In rats PPAR*δ* mRNA is three times higher in slow than in fast muscles, and the protein appears to be accumulated in oxidative fibres (Lunde *et al.*, 2007). Swimming exercise increased muscle PPAR*δ* mRNA in rats on a high-fat diet, but had no effect in lean rats (Kannisto *et al.*, 2006). In normally fed mice, PPAR*δ* protein increased significantly after three weeks of moderate treadmill exercise, but short-term effects were not investigated in this study (Luquet *et al.*, 2003). In a human microarray study, the PPAR*δ* signal was found to be elevated during recovery after a single bout of cycling exercise to exhaustion (Mahoney *et al.*, 2005). In rat, the effects of precisely defined activity patterns have been studied by implanting electrodes. When a slow activity pattern was imposed on a fast muscle *via* the nerve, the level of PPAR*δ* mRNA trebled in 24 h. In order to study the effect of a fast pattern on slow muscles, the muscles had to be denervated to abolish slow activity. While denervation itself had little effect on the level of PPAR*δ* mRNA, a fast stimulation pattern reduced the level to less than half within 24 h (Lunde *et al.*, 2007). Since only a limited group of muscles below the knee in one leg were stimulated in otherwise normally active rats, and since the effects were compared to contralateral muscles, electrical activity apparently regulates PPAR*δ* levels locally and not as a consequence of increased overall physical activity. A possible mediator of the activity effect is calcineurin, thus expression of PPAR*α*, PPAR*δ* and the PPAR cofactor PGC-1*α* were promoted by expression of a constitutively active form of calcineurin in both fast and slow muscles in transgenic mice (Long *et al.*, 2007).

In transgenic mice, where the PPAR*δ* gene was knocked out in muscle by *α*-actin driven Cre recombinase expression, decreased levels of oxidative enzymes, and a slow-to fast-shift in MyHC and troponin I isoenzymes were observed (Schuler *et al.*, 2006). Transgenic mice overexpressing wild-type PPAR*δ* selectively in skeletal muscle develop muscles with elevated oxidative capacity and some hyperplasia, but show no alterations in MyHC fibre type (Luquet *et al.*, 2003). When a ligand-independent constitutively active VP16–PPAR*δ* fusion protein was expressed, however, the transgenic animals also developed muscles with more type I fibres (Wang *et al.*, 2004*b*). It is tempting to speculate that the inability of the wild-type PPAR*δ* to induce MyHC fibre-type change is due to lack of ligand in muscles lacking slow activity. However, the transgenic technology also differed between the experiments; in the mice expressing the wild-type protein probably only some of the muscle nuclei were expressing the transgene. Nonetheless, the findings may indicate that a moderately elevated level of PPAR*δ*-activity during late muscle development induces muscles to become more oxidative, while with higher activity, MyHC fibre type is also changed. This might be reminiscent of the effects of moderate and hard training in adults but based on the transgenic model developmental, rather than adult effects cannot be excluded.

In adult rats overexpression of PPAR*δ* after electroporation showed that constitutively active VP16-PPAR*δ* can induce both oxidative metabolism and changes in fibre type in adult muscle. In the fast EDL muscle the number of IIa fibres was almost doubled, mainly at the cost of IIb fibres. In all of the transfected fibres, irrespective of type, the level of the oxidative enzyme succinate dehydrogenase (SDH) was increased (Lunde *et al.*, 2007).

These experiments suggest that active PPAR*δ* can serve as a changing agent in adult muscle, but they do not illuminate the role of its ligands. Treatment with the PPAR agonist GW501516 up-regulates mRNA for slow fibre contractile proteins, promotes mitochondrial biogenesis (Wang *et al.*, 2004*a*), and works synergistically with exercise so as to increase endurance (Narkar *et al.*, 2008). Further experiments are needed to determine the role of lipid ligands in muscle plasticity.

In conclusion, the level of PPAR*δ* is up-regulated by slow activity and down-regulated by fast activity. Activity can increase the PPAR*δ* signal both by increasing the protein level and by activating ligands. An increased PPAR*δ* protein level would increase the sensitivity of the muscle to lipids created by activity. In addition PPAR*δ* might be regulated by its co-factors PGC-1*α* and—*β* as discussed above and in Fig. 4.

#### (c) Oxygen

Intracellular oxygen pressures (*P*_O2i_) have been investigated using ^1^H magnetic resonance spectroscopy of myoglobin. *P*_O2i_ are reduced from >27 hPa at rest, to values lower than 5 hPa during exercise (Richardson, Newcomer & Noyszewski, 2001; Richardson *et al.*, 1995). These numbers are averages, and the importance of variability in fibre type was not assessed (Hoppeler *et al.*, 2003). In animals exposed to chronic hypoxia in general a slow-to-fast transformation is observed (Bigard *et al.*, 2000; Faucher *et al.*, 2005; Itoh *et al.*, 1990), but this could be related to changes in activity, and the change can be partly counteracted by exercise. There are however well-characterized signaling pathways sensing oxygen levels.

Prolyl hydroxylases use molecular oxygen as a substrate and hence can act as oxygen sensors (Fig. 4). Specifically HIF-1*α* prolyl hydroxsylases (HPHs) affect the stability of hypoxia inducible factor 1*α* (HIF-1*α*) by hydroxylation of conserved prolines. Under normoxic conditions prolyl hydroxylation enables the interaction of HIF-1*α* and the von-Hippel-Lindau protein, subsequent ubiquitination and proteasomal degradation; under hypoxic conditions HIF-1*α* accumulates in the cells and acts as a transcription factor (Bracken, Whitelaw & Peet, 2003).

It is possible that HIF-1*α* may work on fibre-type-specific genes. The level of HIF-1*α* has been shown to be higher in fast/glycolytic than in slow/oxidative muscles, and the level was increased after hind limb suspension where the muscles become more glycolytic (Pisani & Dechesne, 2005). A HIF-1*α* knock-out mouse displayed a shift towards a more oxidative metabolism (Mason *et al.*, 2004), but there were no shifts in fibre-type composition (Mason *et al.*, 2007). This could however be due to compensatory mechanisms during development. We have observed that when HIF-1*α* was overexpressed in adult rat muscle a shift in the fast direction was observed both in the soleus and the EDL. In the soleus, which contains mainly type I fibres, the number of faster IIa fibres was trebled and in the fast EDL the number of IIb fibres was increased by about 50% at the expense of the slower IIa and IIx fibre types in this muscle (K. Gundersen and I.G. Lunde, unpublished data).

The finding that Hif-1*α* induced a slow-to-fast transformation in fibre type was not necessarily predicted, since most forms of exercise activity might reduce muscle *P*_O2i_. The *P*_O2i_ experienced by individual muscle fibres would however be a complex function of oxygen supply and consumption: supply will vary between richly capillarized oxidative fibres and poorly vascularized glycolytic fibres. On the other hand, oxidative fibres use more oxygen than glycolytic fibres for the same amount of energy consumption. In addition, the pattern of activity varies between fibre types, and intramuscular pressure might restrict blood flow during high-intensity contraction. Adding to the complexity is the observation that the temporal aspects of hypoxia matter, thus intermittent hypoxia has been shown to be more efficient in accumulating HIF-1*α* than chronic hypoxia (Semenza, 2009).

Although precise information on the *P*_O2i_ in different muscle fibre types during various activity conditions is not available (Hoppeler *et al.*, 2003), there are indications that fast/glycolytic fibres experience a more severe hypoxia during contraction than do slow/oxidative fibres (McDonough *et al.*, 2007). From a teleological point of view it might be argued that it is beneficial if muscles that experience hypoxia become more glycolytic and use less oxygen, or if severe hypoxia is related to high-intensity exercise, that fibres used in such become faster, stronger and more glycolytic to meet the demand. In sports physiology, the possibility of hypoxia acting as a changing agent for muscle also has interesting implications for altitude training or training with obstructed blood supply, and for understanding muscle changes correlated with respiratory disease.

### (3) Six1 and eya1

The signaling molecules discussed above can easily be placed in a general signaling pathway such as outlined in Fig. 3. For sine oculis homeobox 1/eyes absent 1 (Six1/Eya1) and muscle TFII-I repeat-domain-containing protein 1 (MusTRD; see Section III.4) such placement is perhaps more difficult, and it is inspiration from developmental biology that has led to suggestions that these transcription factors might play a role in muscle plasticity.

The Six-family of transcription factors was established by structure homology to its first member the sine oculis gene (*so*) in *Drosophila melanogaster*. In mice the six-family has five member-genes (*Six1–5*). The factors have been implicated particularly in the development of eye and muscle. Six proteins seem to bind to ARE and MEF3 binding motifs of promoters (Kawakami *et al.*, 2000). Eya is a metal-dependent protein tyrosine phosphatase that can act both as a transcription factor and an enzyme. In mammals there are four paralogs (Eya1-4). The role of Eya as a phosphatase is still unclear, but interestingly it has both itself and MAPK as substrates, and it is also itself a MAPK substrate (Hsiao *et al.*, 2001; Jemc & Rebay, 2007). The activation of Eya by MAPK may provide a link to fast activity (Fig. 4).

Eya itself lacks a DNA binding motif and is considered a transcription co-factor. Eya interacts physically with Six, and although both Eya and Six proteins alone promote low levels of transcription, co-transfection studies have produced robust synergetic activation. Six seems to be responsible for accumulating Eya in cell nuclei where the complex can act as a transcription factor (Jemc & Rebay, 2007).

In zebrafish (*Danio rerio*) a Six1 homolog is essential for development of fast muscle fibres (Bessarab *et al.*, 2008). Six1, −4 and −5 are expressed in vertebrate adult skeletal muscle (Spitz *et al.*, 1998). Surprisingly Six1 mRNA was not accumulated in fast muscles, but western blotting of total protein fractions revealed protein accumulation. Similarly, Eya1, −2 and −4 were all expressed at the mRNA level in muscle, but judged by immunohistochemistry only Eya1 protein was accumulated in nuclei of fast fibres (Grifone *et al.*, 2004).

When Six1 and Eya1 were overexpressed in adult soleus, products of endogenous fast genes such as MyHCIIb, MyHCIIx, MyLC3f, SERCA1 and the glycolytic enzyme *β*-enolase were induced. Moreover, MyHCI, MyHCIIa and the oxidative enzymes SDH and nicotine adenine dinucleotide tetrazolium reductase (NADH-TR) were supressed (Grifone *et al.*, 2004). Thus, Six1/Eya1 seem to induce most of the phenomena connected to a slow-to-fast transformation such as induction of fast genes, inhibition of slow genes, and a shift from oxidative to glycolytic metabolism.

Interestingly blocking calcineurin with FK506 did not lead to accumulation of Six1 or Eya1 (Grifone *et al.*, 2004). Since calcineurin is believed to mediate effects of slow activity, this suggests that Six1/Eya1 is not activated as a default in absence of slow activity, but as a specific fast mechanism. During development it has been demonstrated that Six1 might be a target gene for myogenic factors. In particular MyoD is strikingly efficient at activating Six1 (Ishibashi *et al.*, 2005). If similar mechanisms operate in the adult, MyoD may be linking Six1 levels to fast activity (Fig. 4).

Six1/Eya1 might also work by regulating the MRFs, as during muscle development, Six1 seems to be implied in regulating all four of them (Giordani *et al.*, 2007; Grifone *et al.*, 2005; Spitz *et al.*, 1998; Zhang & Stavnezer, 2008), but it remains to be seen if Six1/Eya1 regulates myogenic factors in the adult.

There is evidence that Six1/Eya1 could act directly by binding to relevant promoters. When Six1 and Eya1 were co-electroporated with reporter-promoter constructs for the glycolytic enzyme aldolase A, the promoter was activated. Reporter levels increased both when the transgene is episomal and when incorporated into the genome. Thus, in transgenic promoter-reporter mice, reporter was about 10-fold higher in fast than in slow muscle, and mutating the MRF3 site reduced expression in fast muscle, but had no effect on slow muscle (Grifone *et al.*, 2004). Electromobility shift assays (EMSA) demonstrated that the Six1/Eya1 complex binds directly to a MEF3 binding site in the aldolase A promoter, and a trimerized MRF3 site from the promoter is transactivated by Six1/Eya1 *in vivo*. Thus, Six1/Eya1 might together with the inhibitory effect of NFAT on the fast troponin I promoter, be the best-documented example of an ultimate transcription factor involved in differential expression of fibre-type-specific genes.

### (4) MusTRD

Muscle TFII-I repeat-domain-containing protein 1 (MusTRD1) is a protein with homology to the transcription factor TFII-1, and was cloned as a protein binding to an element within the SURE region of the troponin I promoter (O'Mahoney *et al.*, 1998). MusTRD has been cloned independently by others and been given a variety of names such as GTF3, WBSCR11, GTF2IRD, CREAM and BEN. MusTRD is coded by a gene termed *GTF21RD1* and is expressed in several splice forms with different DNA-binding abilities (Issa *et al.*, 2006; Polly *et al.*, 2003; Vullhorst & Buonanno, 2003). MusTRD mRNA was originally described as expressed predominantly in muscle (O'Mahoney *et al.*, 1998), but others have found that it is ubiquitously expressed (Bayarsaihan & Ruddle, 2000; Calvo *et al.*, 2001). MusTRD is found in high levels in C2C12 tissue culture and in developing or regenerating muscle, but the level in muscle decreases sharply between postnatal day 7 and 15. During development there is no difference between fast and slow muscle, although in the adult it is marginally higher (<1.4-fold) in the slow soleus compared to the fast EDL (Calvo *et al.*, 2001). Taken together, 11 different splice forms have been found in C2C12 cells, and in developing and adult muscle. Although some of the splice variants were differentially expressed spatially in animals, a fibre-type-specific expression was not reported (Tay *et al.*, 2003). A binding site for MusTRD has been demonstrated in the troponin I promoter by gel shift assays (Calvo *et al.*, 2001; O'Mahoney *et al.*, 1998), and when MusTRD was overexpressed after electroporation in adult muscle, expression from a SURE-luciferase promoter reporter construct was repressed (Calvo *et al.*, 2001). This was essentially confirmed in COS cells by studying a trimerized human slow troponon I binding site connected to luciferase. It was suggested, however, that MusTRD could have a suppressive effect without binding to the SURE promoter since a MusTRD lacking the DNA binding domain also suppressed expression of the reporter. It was suggested that the indirect effect could be due to MusTRD physically interacting with nuclear receptor co-repressor (NoCR), or by interfering with MEF2 binding (Polly *et al.*, 2003).

Recently, transgenic mice overexpressing MusTRD in muscle *via* the *α*-actin promoter were created. Although transgenic transcripts were detected already at 13.5 days *post coitum* the animals were born with muscles that had normal mass, fibre number and fibre type, however they displayed kyphoscoliosis. During the first few post-natal weeks the animals lost their type I fibres, in what appeared to be a type I to IIa transformation in the soleus. Most other slow isoenzymes were also lost, including slow troponin I but not myosin light chain 2 (MyLC2) and oxidative enzymes (Issa *et al.*, 2006). The change in fibre type coincided with the time when distinct fast and slow adult nerve-evoked activity patterns are established (Buffelli *et al.*, 2002; Personius & Balice-Gordon, 2001; Vrbova, Navarrete & Lowrie, 1985), so it is tempting to suggest that MusTRD is involved in activity regulation. In adults, however, an active role of MusTRD is hard to reconcile with its low protein levels, and the lack of differential expression in fast and slow muscle. However the reduced level of MusTRD may be crucial for maintaining slow fibre properties in the adult.

In conclusion, it is unclear if MusTRD plays any role in adult muscle plasticity. Its role might be purely developmental, in playing a restrictive role for development of fibre type prenatally; in addition its absence seems crucial for maintaining normal fibre-type distribution after birth. The complex effects of MusTRD underscore the notion that factors might have different roles at different developmental stages.

## IV. PLASTICITY OF MUSCLE FORCE

### (1) The cell biology of muscle fibre size

**Fig. 5:**
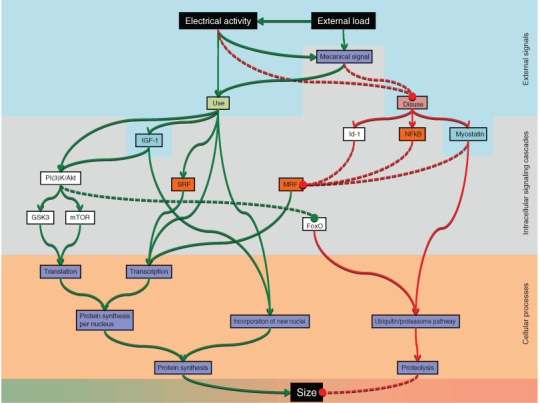
Diagram summarizing pathways currently believed to be involved in regulating muscle fibre size. The pathways have different degrees of scientific support, and their relative quantitative importance is still poorly understood. For key to line and box types, see key to Fig. 4. Abbreviations: forkhead box O (FoxO), glycogen synthase kinase 3 (GSK3), inhibition-of-DNA-binding-protein 1 (Id-1), insulin-like growth factor I (IGF-1), mammalian target of rapamycin (mTOR), myogenic regulatory factor (MRF), phosphatidylinositol 3-kinases/Akt (PI(3)K/Akt), serum response factor (SRF), *κ* light polypeptide gene enhancer in B-cells (NF*κ*B).

Regulation of force is mainly a question of regulating fibre size, and ultimately size is regulated by altering the balance between protein synthesis and degradation in each muscle fibre. As illustrated in the lower part of Fig. 5, change in fibre size can be achieved by regulating three major conditions: (1) the number of nuclei; (2) the rate of protein synthesis for each nucleus; and (3) the rate of protein degradation.

With regard to the regulation of the number of nuclei, it has long been thought (Strassburger, 1893) that a nucleus can support a certain volume of cytoplasm, and that a constant myonuclear domain is maintained during changes in fibre size. Thus, it has generally been believed that hypertrophy was accompanied by addition of new nuclei by satellite cells fusing with the pre-existing muscle fibre syncytia (Lipton & Schultz, 1979; Moss & Leblond, 1970), and that atrophy was accompanied by loss of myonuclei by a selective apoptosis of some nuclei within the intact fibres (Adhihetty *et al.*, 2007; Allen *et al.*, 1997*a*,*b*; Alway *et al.*, 2003*a*,*b*; Borisov & Carlson, 2000; Darr & Schultz, 1989; Dupont-Versteegden *et al.*, 2000, 1999, 2006; Jin *et al.*, 2001; Schmalbruch & Lewis, 2000; Siu & Alway, 2005; Siu, Pistilli & Alway, 2005*a*; Siu *et al.*, 2005*b*; Tang *et al.*, 2000; Tews, 2005; Tews *et al.*, 1997; Viguie *et al.*, 1997; Yoshimura & Harii, 1999).

As reviewed previously, recent research suggests that the nuclear domains may be less constant than previously thought (Gundersen & Bruusgaard, 2008). Moreover, although apoptosis is commonly observed in atrophying muscle tissue, myonuclei seem to be excluded from apoptosis under atrophy conditions. Time-lapse imaging indicates that the number of nuclei is not altered during atrophy, thus nuclei are not lost by a selective intracellular apoptosis as suggested previously (Bruusgaard & Gundersen, 2008; Gundersen & Bruusgaard, 2008).

The finding that nuclear domains may not be as tightly regulated as previously thought (Gundersen & Bruusgaard, 2008), and the lack of elimination of myonuclei during atrophy observed by *in vivo* imaging (Bruusgaard & Gundersen, 2008) also questions the idea that myonuclei are added to muscle fibres during hypertrophy. The issue of whether activation and fusion of satellite cells is obligatory for hypertrophy was recently debated in the *Journal of Applied Physiology* (Bodine, 2007; Booth, 2007; Hikida, 2007; Kadi, 2007; Lowe, 2007; Mantilla & Sieck, 2007; McCarthy & Esser, 2007; O'Connor & Pavlath, 2007; O'Connor *et al.*, 2007; Rehfeldt, 2007). It was argued that molecular evidence suggests that there is an early increase in protein synthesis (per nucleus), and that addition of nuclei follow later if at all. Based on *in vivo* imaging we have observed a different temporal picture at the phenomenological level. When mice EDL muscle was overloaded by synergist ablation the addition of new nuclei occurred mainly after 6–8 days, while the increase in size occurred after 8–12 days (Bruusgaard *et al.*, 2010). Thus addition of nuclei preceded the hypertrophy leading to a temporary decrease in myonuclear domain sizes. This sequence of events strongly suggests that the increased number of nuclei is a major cause of hypertrophy, but it does not preclude that at least some hypertrophy could occur in the absence of new nuclei under experimental conditions where satellite cells are eliminated.

Interestingly, newly added “hypertrophy nuclei” seem to be maintained during subsequent atrophy. Thus, the elevated number of nuclei was maintained even after three months of denervation resulting in severe atrophy (Bruusgaard *et al.*, 2010). This might explain the phenomenon of “muscle memory”: that previously strong muscles seem to be more easily retrained. The number of nuclei might be limiting during the first hypertrophy, and the increased number of nuclei resulting from hypertrophy might facilitate increased synthesis upon retraining.

### (2) Paracrine and autocrine mechanisms

While the regulation of protein synthesis per nucleus and protein degradation in principle could be obtained by an intracellular excitation-transcription coupling such as discussed for regulation of fibre type; involvement of satellite cells would require additional intercellular signaling systems. Interestingly, there seem to be at least two systems acting as local hormones in muscle, contributing to regulation of muscle fibre size in the adult: myostatin and IGF-1. The release of these factors is dependent on activity (upper part of Fig. 5).

#### (a) Myostatin

Myostatin (previously called GDF-8) is a member of the transforming growth factor *β* (TGF-*β*) superfamily and plays a major role during development where it acts as an inhibitor of muscle growth. Disruption of the myostatin gene leads to development of grossly enlarged muscles in mice (McPherron, Lawler & Lee, 1997), farm animals (McPherron & Lee, 1997), and man (Schuelke *et al.*, 2004). The enlargement is caused both by an increase in the number of fibres (hyperplasia) and in fibre size (hypertrophy). Importantly, muscle enlargement obtained by myostatin deficiency is peculiar because it does not increase force in proportion to size, thus the amount of contractile proteins may not be properly regulated (Amthor *et al.*, 2007; Bruusgaard *et al.*, 2005). Thus, reducing myostatin alone might not mimic effects of strength training, although strength training in adults has been shown to be associated with reduced levels of myostatin in muscle and plasma (Heinemeier *et al.*, 2007; Kim, Cross & Bamman, 2005; Roth *et al.*, 2003; Walker *et al.*, 2004). Conversely various disuse conditions have been associated with elevated levels (Gonzalez-Cadavid *et al.*, 1998; Lalani *et al.*, 2000; Reardon *et al.*, 2001; Wehling, Cai & Tidball, 2000; Yarasheski *et al.*, 2002). Interestingly, release of myostatin can be regulated by the titin-cap protein (Nicholas *et al.*, 2002), opening up the possibility that stretch signals could regulate myostatin release.

In adult animals inhibition of myostatin with antibodies leads to hypertrophy without an increase in the number of fibres (Bogdanovich *et al.*, 2002); conversely, overexpression of myostatin in muscle fibres after electroporation leads to muscle atrophy without loss of muscle fibres (Durieux *et al.*, 2007). In the latter study it was suggested that myostatin acted by reducing muscle gene expression of myofibrillar proteins perhaps by reducing expression of MyoD and myogenin. In addition, myostatin might activate the ubiquitin-proteasome pathway for proteolysis (Gilson *et al.*, 2007).

#### (b) Insulin-like growth factor I (IGF-1)

IGF-1 has been implicated as a factor promoting hypertrophy in the adult animal. The gene gives rise to several proteins by differential splicing with a rather confusing nomenclature (Barton, 2006*a*). Adding to the complexity is the observation that IGF-1 activity is regulated by IGF-binding proteins acting as carriers in blood, or locally binding IGF-1 to the extracellular matrix (Clemmons, 1998, 2007).

The liver supplies approximately 75% of the circulating IGF-1 (Schwander *et al.*, 1983), and a selective abolishment of IGF-1 production in hepatocytes leads to a 75% reduction in circulating IGF-1 levels but without growth impairment (Sjogren *et al.*, 1999). In humans increasing the circulating level of IGF-1 does not promote muscle protein synthesis (Yarasheski *et al.*, 1992, 1993). IGF-1 is also expressed locally in several tissues including muscle where it is induced by stretch or high-resistance exercise (Adams & Haddad, 1996; Adams, Haddad & Baldwin, 1999; Bamman *et al.*, 2001; DeVol *et al.*, 1990; Petrella *et al.*, 2006; Sakuma *et al.*, 1998; Singh *et al.*, 1999; Yan, Biggs & Booth, 1993). A specific splice variant of IGF-1 is particularly prone to be up-regulated under such conditions, and has been dubbed mechano growth factor (MGF) (Hameed *et al.*, 2003; Yang *et al.*, 1996). Muscle also produces IGF-1 during exercise (Brahm *et al.*, 1997), although plasma IGF-1 levels are not significantly altered (Walker *et al.*, 2004). Based on these findings, it is suggested that IGF-1 production is triggered by resistance exercise, and can act in a para- and autocrine fashion in muscle.

Transgenic mice over-expressing IGF-1 have increased muscle mass (Mathews *et al.*, 1988) while mice deficient in IGF-1 have a severe muscular dystrophy (Powell-Braxton *et al.*, 1993). Also transgenic mice overexpressing IGF-1 selectively in muscle cells display muscle hypertrophy (Coleman *et al.*, 1995; Musaro *et al.*, 2001). In adult muscles, localized infusion of IGF-1 protein increased muscle mass (Adams & McCue, 1998), and muscle transfected with expression vectors for IGF-1 by electroporation (Alzghoul *et al.*, 2004) or viral infection (Barton, 2006*b*) displays hypertrophy. These findings suggest that IGF-1 plays a role in adult hypertrophy.

The selective effects of different splice forms have been investigated. In muscles of two-week-old animals where muscles were infected with IGF-1-producing adenoviruses, hypertrophy was observed with both the MGF (IGF-1b) and IGF-1a isoforms, but only the IGF-1a form had effects when introduced in mature mice (Barton, 2006*b*).

In muscle tissue culture, IGF-1 has been shown to increase myotube diameter, suppress proteolysis, stimulate protein synthesis, and induce a higher number of nuclei per length of myotube (Engert, Berglund & Rosenthal, 1996; Florini, Ewton & Coolican, 1996; Jacquemin *et al.*, 2004; Rommel *et al.*, 2001; Vandenburgh *et al.*, 1991). IGF-1 seems to promote myoblast mitosis as well as myoblast differentiation (Rosenthal & Cheng, 1995). Thus IGF-1 might lead to production of new myoblasts, and promote them to fuse to existing myotubes. In addition, IGF-1 has a direct effect on protein synthesis mainly by stimulating translation (Philippou *et al.*, 2007).

*In vivo* IGF-1 increases the DNA content in muscles (Adams & McCue, 1998), but it remains to be demonstrated if IGF-1 induces satellite cells to fuse to pre-existing fibres. Elevated IGF-1 immunoreactivity has been demonstrated in myoblasts and myotubes during muscle regeneration (Jennische & Hansson, 1987; Jennische, Skottner & Hansson, 1987), and a loss-of-function study with an IGF-1 antibody showed that the number and size of regenerating muscle fibres is reduced (Lefaucheur & Sebille, 1995).

In conclusion, IGF-1 seems to work as a local hormone that promotes hypertrophy in adult muscle. It might do so both by interfering with protein balance in muscle fibres and by activating satellite cells, but for the latter there is still little information in adult muscles.

### (3) Intracellular pathways of size regulation

A discussion on signaling pathways for atrophy and hypertrophy can easily be rather complex since it involves signaling both inside and between at least two different cell types: satellite cells and myofibres. As discussed above, multiplication, differentiation and the ultimate fusion of satellite cells to pre-existing muscle fibres is thought to be important for hypertrophy, but satellite cell behaviour and regulatory pathways have been mostly studied during muscle formation and regeneration (Holterman & Rudnicki, 2005; Philippou *et al.*, 2007; Wagers & Conboy, 2005; Zammit, 2008), and less so in intact adult muscles.

#### (a) Regulation of protein production in muscle fibres

##### (i) Regulation of transcription

During changes in muscle fibre size, protein production seems to be regulated both at the transcriptional and at the translational level (for review see Favier, Benoit & Freyssenet, 2008). It is tempting to suggest that transcription factors operating during development also act in the adult. For example the developmentally important MRFs have been implicated also in regulation of fibre size, and can be up-regulated by resistance exercise. Conversely Id proteins are up-regulated during inactivity (Alway *et al.*, 2003*b*; Carlsen & Gundersen, 2000; Dupont-Versteegden *et al.*, 1998; Gundersen & Merlie, 1994). When Id-1 was overexpressed, it induced atrophy in a dose-dependent manner (Gundersen & Merlie, 1994). However also the MRFs themselves (Buonanno *et al.*, 1992; Duclert, Piette & Changeux, 1991; Eftimie, Brenner & Buonanno, 1991; Merlie *et al.*, 1994; Neville, Schmidt & Schmidt, 1992; Walters, Stickland & Loughna, 2000*b*; Witzemann & Sakmann, 1991), and their positive partners; the E-proteins (Carlsen & Gundersen, 2000), are up-regulated during disuse. The putative role of the MRFs is illustrated in Fig. 5, but more research is required to investigate the role of MRFs, their phosphorylation state, and their partners in order to establish their importance for transcriptional regulation during atrophy/hypertrophy conditions in the adult.

The transcription factor serum response factor (SRF) is increased by stretch in avian muscle, and seems to bind to and transactivate the alpha actin gene (Carson & Booth, 1999; Carson, Schwartz & Booth, 1996; Fluck *et al.*, 1999). If SRF is a major regulator of this important contractile protein, SRF might have a central role in regulating fibre size.

It has been suggested that the transcription factor NF*κ*B contributes to muscle atrophy, activated by the cytokine tumor necrosis factor *α* (TNF-*α*) which is activated in disease states, leading to atrophy (Kandarian & Jackman, 2006; Tisdale, 2009). Disuse atrophy does not however, seem to trigger TNF-*α* production, but the concentration of NF*κ*B is increased by unloading (Hunter *et al.*, 2002). NF*κ*B may downregulate MyoD and hence synthesis of muscle-specific proteins (Guttridge *et al.*, 2000).

##### (ii) Regulation of translation: the PI(3)K/Akt pathway

An important regulated process is translation. During hypertrophy, IGF-I seems to activate the phosphatidylinositol 3-kinases/Akt/mammalian target of rapamycin (PI(3)K/Akt/mTOR) pathway, which seems to boost translation (Bodine *et al.*, 2001*b*; Li, Li & Parkhouse, 2002; Rommel *et al.*, 2001). This pathway is illustrated in Fig. 5, and has been reviewed extensively (Adams, 2002; Favier *et al.*, 2008; Miyazaki & Esser, 2009; Sandri, 2008). mTOR seems to be crucial, since selective mTOR blockers inhibit hypertrophy *in vivo*. Interestingly such inhibition only prevented use-related hypertrophy, but did not induce atrophy (Bodine *et al.*, 2001*b*).

*In vitro* evidence suggests, that there is also an alternative collateral mTOR-independent pathway where Akt activates glycogen synthase kinase 3 (GSK-3) (Fig. 5) (Holterman & Rudnicki, 2005; Rommel *et al.*, 2001), but since the results might be confounded by effects on muscle differentiation *in vitro,* more research on this pathway should be performed *in vivo* in order to establish its importance for muscle plasticity.

The PI(3)K/Akt/mTOR pathway seems to operate in mice expressing a dominant negative IGF-1 receptor in muscle (Spangenburg *et al.*, 2008), suggesting that overload hypertrophy is at least not solely dependent on activation of the IGF-1 receptor, and that the pathway can be activated also by other signals.

In addition to its effects on translation, the PI(3)K/Akt pathway inhibits FoxO, which is an activator of the ubiquitin-proteasome pathway, and thus protein degradation (Glass, 2005).

#### (b) Regulation of protein degradation

Atrophy is caused both by reduced protein production, and by increased proteolysis. The latter has received most of the attention recently, partly because the specific mechanisms are easier to study, but it has also been claimed that it is quantitatively the most important process during atrophy (Kandarian & Jackman, 2006). The large body of literature on the regulation of protein degradation in muscle during atrophy has been extensively reviewed (Favier *et al.*, 2008; Glass, 2003, 2005; Kandarian & Jackman, 2006; Sandri, 2008), and the pathways will be only briefly outlined here.

The central pathway for proteolysis during atrophy is the ubiquitin-proteasome pathway. With this mechanism doomed proteins are “tagged” by multiple copies of a 76-amino-acid peptide, called ubiquitin, being ligated to a lysine residue. These ubiquitinated proteins are then degraded by a large protein complex called the 20 S proteasome (Jung, Catalgol & Grune, 2009). The ubiquitination is accomplished by a complex enzymatic reaction where members of a large group of ubiquitine-ligases participate, each one specific to a limited number of substrate proteins (Jung *et al.*, 2009). Two such proteins have been shown to be up-regulated in at least 13 distinct atrophy models: muscle ring finger1 (MuRF1) and muscle atrophy F-box protein (MAFbx, also called atrogin) (Glass, 2005). Mice with null-mutations for one of these proteins appear normal, but atrophy is attenuated (Bodine *et al.*, 2001*a*). MuRF1 has also been shown to inhibit nuclear localization and transcriptional activity of SRF (Lange *et al.*, 2005), and may thus contribute to reduced transcription during atrophy.

#### (c) Putative mechano-sensing mechanisms in muscle size regulation

Interestingly, MuRF1 has been shown to bind specifically to titin (Centner *et al.*, 2001; Lange *et al.*, 2005; McElhinny *et al.*, 2002; Mrosek *et al.*, 2007), and it has been postulated that titin may serve as a mechanosensor relaying stretch signals to intracellular pathways. Titin appears to contain a kinase domain that binds Ca^2+^/calmodulin (Mayans *et al.*, 1998). The domain is located in the M-line, a position that would be prone to stretch during active muscle contraction, and recent experiments combining atomic force microscopy, molecular dynamics simulations, and enzymatics suggest that the catalytic domain is exposed by stretch (Puchner *et al.*, 2008). This makes titin a prime force-transducer candidate (Tskhovrebova & Trinick, 2008). In cardiomyocytes, an elaborate mechano-sensitive signaling system connected to the contractile apparatus and mediating stretch signals has been postulated (for review see Kruger & Linke, 2009), but its relevance to skeletal muscle is still unclear.

## V. THE EXCITATION-TRANSCRIPTION COUPLING: TOWARDS A SYNTHESIS?

### (1) Plasticity of speed

Nadal Ginard has termed the factor linking activity and adult muscle phenotype “the holy grail” of muscle biology. As evident from Fig. 4, there seems to be a large number of such grails, with few simplifying principles. It is a problem that we currently do not have an understanding of the relative importance of different pathways, but it is possible that more loss-of-function experiments could reveal which are the major pathways that are crucial for plasticity.

Several activity correlates might be sensed by various sensor molecules such as HPH, PKC, calmodulin, AMPK, and PPAR*δ*. The activation of these sensors triggers complex cascades ending up with activation or inhibition of ultimate transcription factors binding to promoter regions of fast and slow genes. Most of the research has been on participants in cascades, and since they interact in complex ways and also might interfere with fundamental and general cell biological mechanisms; it is often difficult to assess their specific importance for muscle plasticity. It is possible that more emphasis should be put on unraveling signaling pathways from the distal end: which are the factors binding to the promoters for fast and slow isoforms?

A hard-earned lesson from the study of gene regulation is that the mechanisms seem to be different in different experimental systems. Thus, cultured, developing or regenerating muscles are different from adult muscles, and endogenous genes frequently behave differently from promoter-reporter constructs. Although inspiration might be drawn from other systems, ultimately any hypotheses would have to be tested on endogenous genes in adult muscle that have developed in the absence of genetic manipulation.

Based on experiments in developing or regenerating muscle, it has been suggested that there are pathways connecting slow activity to slow phenotype, but that fast phenotype develops as a default in the absence of slow activity (Butler-Browne *et al.*, 1982; Esser, Gunning & Hardeman, 1993; Jerkovic *et al.*, 1997). This idea of a default fast pathway is not supported in adult muscle. Thus, slow phenotype can develop in anural muscles (Condon *et al.*, 1990; Phillips & Bennett, 1984), and in adult muscle, absence of activity does not lead to the same phenotype as stimulation with a fast pattern. Not only does a denervated or inactive muscle become extremely atrophic, but it also displays abnormal membrane properties such as supersensitivity for acetylcholine. This abnormality is not seen in denervated muscles given even very little activity (Lømo & Westgaard, 1975; Midrio, 2006). After denervation, the twitch at least initially becomes slower (Gundersen, 1985), and even if fast MyHC is eventually induced in a minority of fibres, fast stimulation will induce fast MyHC in all fibres (Windisch *et al.*, 1998). Promoter-reporter studies *in vivo* showed that denervation inhibited promoters for both the fast and slow troponin I isoforms, while the fast promoter was selectively activated by a fast pattern and not a slow pattern (Rana *et al.*, 2008, 2005). These findings suggest that there is a specific fast signaling pathway, at least for troponin I. The idea of a default fast pathway might be more relevant for MyHC regulation where a frequency effect has not been demonstrated. A slow activity pattern will phosphorylate and inactivate MyoD, and MyoD protein was shown to trigger expression of fast MyHC only in the absence of externally triggered activity in denervated slow muscles (Ekmark *et al.*, 2007). It was argued that MyoD maintains fast properties in fast muscles where slow activity will not phosphorylate and inactivate it, and this could be an example of a fast signal acting only in the absence of a slow activity pattern.

### (2) Plasticity of force

It is generally accepted that nerve-evoked activity plays a major role in determining muscle size in adults. During activity both electrical and mechanical factors are believed to play a major role in providing signaling (Fig. 5), but there is currently little precise information on the initial mechanisms (activity correlates) involved. Activity seems to act at least partly by altering paracrine conditions in the muscle tissue such as *via* IGF-1 and myostatin.

Our knowledge about pathways further downstream has increased over the last decade, in particular about the intracellular pathways responsible for increased proteolysis during atrophy, and increased translation during hypertrophy. Less is known about transcriptional regulation during such conditions.

The activation of satellite cells is still poorly understood, but it seems clear that fusion of satellite cells seems to play a role during hypertrophy, but contrary to previous ideas myonuclei do not seem to be lost during atrophy. This suggests that myonuclei recruited during strength training are permanent. Thus, effects of strength training might be more permanent than previously thought and the elevated number of nuclei may represent a form of muscle memory facilitating retraining (Bruusgaard *et al.*, 2010).

## VI. CONCLUSIONS

1. Muscle properties change in an adaptive way when the activity pattern delivered to the muscle is changed, and the changes are dependent on the specific fast or slow pattern of activity.
2. Several intracellular changes such as an increase in free intracellular Ca^2+^, alterations in metabolites, hypoxia, and mechanical stress are correlated to muscle activity and molecular sensors connected to intracellular signaling pathways sense various activity correlates and trigger intracellular signaling pathways.
3. The numerous putative signaling pathways reviewed herein as taking part in muscle plasticity rely on scientific support of variable strength. In addition, more quantitative approaches are desirable in order to establish the most important pathways, as opposed to those that have some effects under some experimental conditions. Thus, our current model seems to be in need of both pruning, and new data. To this end loss-of-function studies might be useful.
4. Signaling pathways might involve transcription factors and gene regulation as part of the cascade, but the pathways end up regulating “ultimate” transcription factors binding to the genes for fast and slow isoforms. Very few ultimate transcription factors have been unequivocally identified so far, and more knowledge about such factors might contribute to a more precise unraveling of signaling pathways from the distal end.
5. Mechanisms of adult plasticity should be confirmed by studies of endogenous genes in adult muscle *in vivo*. Insights from other more convenient models, although inspirational, have sometimes proven treacherous.
6. Regulating the balance between protein synthesis and proteolysis alters muscle fibre size. Myonuclei are recruited from satellite cells during hypertrophy, but not lost during atrophy. Thus, the idea of constant myonuclear domains seems oversimplified.
